# Prodrug Approach as a Strategy to Enhance Drug Permeability

**DOI:** 10.3390/ph18030297

**Published:** 2025-02-21

**Authors:** Mateus Mello de Souza, Ana Luísa Rodriguez Gini, Jhonnathan Alves Moura, Cauê Benito Scarim, Chung Man Chin, Jean Leandro dos Santos

**Affiliations:** 1 School of Pharmaceutical Sciences, São Paulo State University (UNESP), Araraquara 14800-903, SP, Brazil; mello.souza@unesp.br (M.M.d.S.); ana.gini@unesp.br (A.L.R.G.); caue.scarim@unesp.br (C.B.S.); chung.man-chin@unesp.br (C.M.C.); 2 Institute of Chemistry, São Paulo State University (UNESP), Araraquara 14800-900, SP, Brazil; jhonnathan.alves@unesp.br; 3 Union of the Colleges of the Great Lakes (UNILAGO), School of Medicine, Advanced Research Center in Medicine (CEPAM), Sao Jose do Rio Preto 15030-070, SP, Brazil

**Keywords:** prodrug, drug permeability, PROTAC, permeability, molecular optimization, drug discovery

## Abstract

Absorption and permeability are critical physicochemical parameters that must be balanced to achieve optimal drug uptake. These key factors are closely linked to the maximum absorbable dose required to provide appropriate plasma levels of drugs. Among the various strategies employed to enhance drug solubility and permeability, prodrug design stands out as a highly effective and versatile approach for improving physicochemical properties and enabling the optimization of biopharmaceutical and pharmacokinetic parameters while mitigating adverse effects. Prodrugs are compounds with reduced or no activity that, through bio-reversible chemical or enzymatic processes, release an active parental drug. The application of this technology has led to significant advancements in drug optimization during the design phase, and it offers broad potential for further development. Notably, approximately 13% of the drugs approved by the U.S. Food and Drug Administration (FDA) between 2012 and 2022 were prodrugs. In this review article, we will explore the application of prodrug strategies to enhance permeability, describing examples of market drugs. We also describe the use of the prodrug approach to optimize PROteolysis TArgeting Chimeras (PROTACs) permeability by using conjugation technologies. We will highlight some new technologies in prodrugs to enrich permeability properties, contributing to developing new effective and safe prodrugs.

## 1. Introduction

The discovery of new drugs is a lengthy, costly, and high-risk process, involving substantial financial investment and a high probability of failure. From 2000 to 2018, the average cost of developing a new drug was estimated at USD 172.7 million, including post-marketing studies. When accounting for failed attempts, the cost rises significantly to USD 515.8 million [1]. Studies indicate that 9 out of 10 approved drugs fail in demonstrating efficacy and safety during various stages of development [2]. The primary reasons for these failures include a lack of clinical efficacy (40–50%), safety concerns due to drug-induced toxicity (30%), inadequate drug-like properties (10–15%), and insufficient strategic planning regarding commercialization and addressing medical needs with an improved efficacy and safety profile compared to existing treatments, when available [3,4].

Following the identification of hits during large-scale systematic screening campaigns, it becomes essential to optimize these compounds to ensure their potency with respect to their molecular target. Additionally, a thorough evaluation of the compounds’ properties, aimed at ensuring adequate tissue exposure, can facilitate a more favorable balance between efficacy, toxicity, and the appropriate dosage. Among the various properties assessed, solubility and permeability are particularly important to maintain an equilibrium, which ensures optimal absorption and exposure to the therapeutic target [3,4].

It has been noted that approximately 40% of marketed drugs, and up to 75% of those in development, exhibit challenges related to low solubility [5]. To address this issue, a variety of strategies have been employed to enhance this important physicochemical property, including particle size reduction [6], the use of advanced pharmaceutical systems (e.g., cyclodextrins [7], lipid-based systems [8], amorphous solid dispersions [9], surfactants [10], and co-solvents [11], among others), as well as the preparation of salts [12], polymorphs [13], co-crystals [14], and the application of prodrug strategies [15]. Among these strategies, the prodrug approach was revealed to be a promising tool to enhance drug solubility [16].

A prodrug is a minimally active or inactive compound that contains a parental drug and undergoes biotransformation in vivo, either through chemical or enzymatic cleavage, facilitating the release of the active molecule at effective levels [17,18]. Bioprecursors and carrier-linked prodrugs represent the primary types of prodrugs. In the case of carrier-linked prodrugs, the carrier can either possess therapeutic activity (as seen in mutual prodrugs) or may not (as in the classical prodrugs) [16,17,18]. Notably, approximately 13% of the drugs approved by the U.S. Food and Drug Administration (FDA) between 2012 and 2022 were prodrugs [18].

Prodrugs play a critical role in addressing solubility and permeability challenges, with advanced design strategies improving bioavailability, stability, and targeted delivery. In addition to improving solubility, prodrugs can also be utilized to modulate drugs’ permeability. A noteworthy analysis conducted by Fralish and colleagues (2024) identified approximately 95 design goals using prodrug strategies, with around 59% of these aimed at enhancing bioavailability, primarily through improvements in permeability (35%) and solubility (15%) [19]. While technologies like cheminformatics and computational modeling have expanded possibilities, challenges such as navigating vast chemical spaces and addressing pharmacogenomic variability remain. Integrating machine learning and pharmacogenomics is essential for advancing safer, more effective therapies.

Membrane permeability is a crucial factor for small molecules to achieve intracellular targets since it is well established that low permeability correlates with low efficacy. In eukaryotic cells, drug permeability across membranes can occur mainly through both active and passive transport mechanisms [20]. Active transport is facilitated by transport proteins that utilize energy (e.g., adenosine triphosphate (ATP) hydrolysis) to assist compounds in crossing the membrane, with the drug’s affinity for the transport protein playing a critical role in this process [21]. However, reliance on transport proteins can result in saturation kinetics due to the limited availability of these proteins within the membrane [21,22]. On the other hand, passive transport does not require energy, as drugs cross the membrane through diffusion. In this case, factors such as the partition coefficient between the membrane and the external aqueous environment, as well as the drug concentration to establish a concentration gradient, contribute to the transport process without energy expenditure. The key factors influencing membrane permeability via passive diffusion include polarity, molecular weight, and lipophilicity [23]. Typically, compounds with lower polarity, smaller molecular weight, and higher lipophilicity (within certain limits) exhibit greater permeability [24].

The estimation of membrane permeability can be assessed using Fick’s first law, expressed as Jr = Pm × Ci, where Jr represents the drug flux rate (mass/area/time), Pm corresponds to membrane permeability, and Ci is the concentration of the drug at the intestinal membrane surface [25,26,27].

The Biopharmaceutical Classification System (BCS) serves as a valuable tool for classifying drugs based on their permeability and water solubility. According to the BCS, drugs are categorized based on solubility and permeability into four main classes [26]: Class I (high solubility and permeability), Class II (low solubility and high permeability), Class III (high solubility and low permeability), and Class IV (low solubility and low permeability) (Table 1) [26,27].

The apparent permeability coefficient (P_app_) is commonly used in in vitro experiments to evaluate the degree of drug permeability between donor and receptor compartments. While the methods used will be detailed later in the text, they are generally correlated with flux between donor and receptor compartments [25,26,27,28,29]. On the other hand, effective permeation (P_eff_) is used to determine in vivo permeability. Although databases for jejunum permeability are well described, information is still missing regarding distal sites (e.g., colon and ileum) in the gastrointestinal tract [30,31]. Both methods present pros and cons and the combination of P_app_ and P_eff_ seems to be a good way to reduce drawbacks [32,33].

Notably, the prodrug approach represents a valuable strategy for modulating membrane permeability [34]. In this review article, we will examine the potential of this approach in addressing challenges related to drug permeability, focusing on drug market and under-developed compounds. New technologies, such as PROteolysis TArgeting Chimeras (PROTACs) and conjugation approaches, will be discussed to provide a comprehensive overview of how prodrugs can be effectively utilized to overcome obstacles in drug optimization and development.

## 2. Permeability Determination Methods

**Figure 1:**
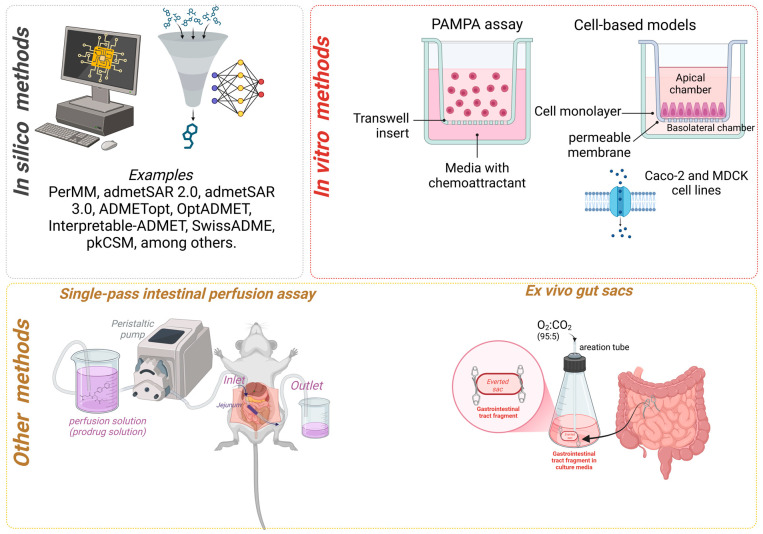
Permeability determination methods. In silico methods: PerMM [35], admetSAR 2.0 [36], admetSAR 3.0 [37], ADMETopt [38], OptADMET [39], Interpretable-ADMET [40], SwissADME [41], pkCSM [42]. (Figure created in https://BioRender.com).

The determination of permeability for prodrugs can be assessed through several methods, which includes in silico, in vitro, cell based, in situ perfusion, ex vivo gut sacs, and ex vivo diffusion chambers (Figure 1). In this context, the general principles of these methods and their application to evaluate prodrug permeability is described below.

### 2.1. In Silico Determination Methods

The assessment of organic compound permeability using computational approaches is an interesting parameter to be characterized during the early phases of drug development, especially for prodrugs. In silico methods facilitate the identification of promising compounds from extensive chemo-libraries in large-scale screening studies and contribute to the molecular optimization process. Some filters such as the "rule of five" are widely used and described as poor permeation and absorption is more commonly found in compounds exhibiting more than five hydrogen bond donors, 10 hydrogen bonding acceptors, molecular weight superior to 500 Da, and calculated logarithm of the octanol/water partition coefficient (logP) values superior to 5 [43]. Based on the number of compounds and the research stage, the knowledge of permeability profile allows for the evaluation of pharmacokinetic and biopharmaceutical properties, playing a crucial role in selecting the most suitable pharmaceutical form and formulation [44].

Computational approaches for assessing permeability via passive diffusion utilize techniques that incorporate lipophilicity, molecular dynamics, and machine learning (ML) [45]. The in silico characterization of a chemical structure’s lipophilicity involves the use of molecular descriptors, such as logP, which represents the logarithmic ratio of the *n*-octanol/water partition coefficient. This parameter is typically regressed against experimental data to enhance the predictive accuracy of the model [46]. LogP can be calculated by using various methods, including the hydrophobic fragmental constant approach (Σf system) for octanol/water partitioning, atom contribution method ALOGP, and element contribution KLOGP [47,48,49]. Another method is the physics-based molecular dynamics (MD) simulations, which are applicable for simulating nanoscale systems and estimating permeability values. For passive permeability, some of the following techniques can be used, depending on the research aim. The potential of mean force and the diffusivity through the membranes allow the calculation of the permeability coefficient (Pe). The most commonly used are the permeant counting method, the homogeneous solubility–diffusion model, and the inhomogeneous solubility–diffusion model [45,50]. Disadvantages of MD simulation that use all-atom MD (AAMD) simulations related to time consumption can be addressed by reducing the degree of freedom and using coarse-grained MD (CGMD) [51,52]. The third approach to estimate the permeability by in silico methods involves the use of artificial intelligence ML methods [53]. The prediction of permeability commonly explores deep neural networks (DNNs) and deep learning (DL) methods. These methods provide values of permeability with high accuracy, and the values can be refined by using experimental data [54]. Chen and colleagues (2020), using the ML model named Least Absolute Shrinkage and Selection Operator (LASSO), reported that the critical descriptors to estimate permeability are hydrophobic/hydrophilic, electro-topological, polarizability, and electrostatic properties [53]. Several examples of web-service for in silico prediction of permeability have been described, including PerMM [35], admetSAR 2.0 [36], admetSAR 3.0 [37], ADMETopt [38], OptADMET [39], Interpretable-ADMET [40], SwissADME [41], and pkCSM [42], among others (Figure 1). These methods are very useful during the drug design stage, and they must be coupled with experimental assays to confirm the results.

In silico methods are widely utilized to evaluate the permeability of prodrugs, demonstrating a satisfactory correlation with experimental data [55,56]. A notable example is used for peptide prodrug design to optimize drug permeability, in which a peptide is linked to a drug through a bio-reversible linker [57]. Although peptide membrane permeability is a common issue faced during drug development, some strategies to improve peptide permeability such as cyclization, amide-to-ester replacement, and *N*-methylation have been shown to be a promising approach. In addition, cell-penetrating peptides (CPPs), a short strand composed of 5–30 amino acids, can overcome barriers such as low drug permeability [58]. In order to determine the permeability of peptides, Tan and colleagues (2024) used a ML model to develop a platform named KNIME (https://github.com/ifyoungnet/PharmPapp) (accessed on 4 January 2025) to assist researchers during a prodrug drug design [59]. Another platform used to predict peptide permeability was developed by Chen and colleagues, named BBP predict (http://i.uestc.edu.cn/BBPpredict/cgi-bin/BBPpredict.pl) (accessed on 4 January 2025), which used ML methods with high accuracy, and is superior to others such as BBPpred and B3Pred [60].

The prediction of the ability of drugs to act on the central nervous system poses a significant challenge due to the semi-permeability and selectivity of the blood–brain barrier (BBB). Conducting clinical experiments to assess BBB permeability is a time-consuming process, and in this context, predictive systems have immense potential to aid in the optimization of compounds and even anticipate the need for the development of specific formulations. Methods for predicting the permeability of compounds in the central nervous system commonly utilize physicochemical descriptors (i.e., molar mass, logP, polar surface area, numbers of hydrogen bond donors and acceptors, ionization potential, and water-accessible and Van der Waals volumes, among others) to evaluate membrane crossing via passive diffusion. However, the complexity of transport systems in substrate recognition, particularly for more hydrophilic compounds, requires more advanced analysis and presents significant challenges [61].

To solve this issue, Gao and colleagues (2022) developed a prediction model that considered both passive diffusion and active transport aiming to predict reposition drugs for central nervous system (CNS) diseases [62]. In the same year, Kumar and colleagues (2022) explored deep learning and ML algorithms for a dataset of 3605 compounds to develop the “DeePred-BBB” model (https://github.com/12rajnish/DeePred-BBB) (accessed on 4 January 2025) with deep learning prediction accuracy of up to 97.6% [63]. BBBper is another example of an ML-based tool available online (http://bbbper.mdu.ac.in) (accessed on 4 January 2025) to predict BBB permeability [63]. The prediction of the ability to cross the BBB was also described by several other platforms elsewhere, especially by using deep learning and ML methods [64,65,66,67].

### 2.2. In Vitro Determination of Permeability

In vitro methods to determine drug permeability involve partitioning among aqueous and lipophilic phases, and such methods can be classified into two main types: artificial membrane systems and cell-based models. One of the most described artificial methods is the Parallel Artificial Membrane Permeability Assay (PAMPA) [68]. The principle of the method is simple and consists of a system divided into two compartments, where the organic compound diffuses via passive diffusion through an artificial membrane. Despite the ease that enables large-scale screening, this method has the drawback of exclusively evaluating passive diffusion processes. For organic compounds that rely on transport systems, inaccurate determinations may occur, and in such cases, cell-based models are required [69].

### 2.3. PAMPA Assay

The passive diffusion method is predominantly assessed using PAMPA assays, with adaptations involving phosphatidylcholine and egg lecithin to establish an additional barrier in the system [70]. It has been reported that the method shows good correlation with permeability in the human jejunum [71]. In general, this method involves preparing a dilution of the test compound in an aqueous solution, which is subsequently added to the donor compartment of a plate. A porous-bottomed 96-well filter plate is positioned atop the donor plate, establishing direct contact with the donor solution. This setup is maintained for varying durations (1–18 h) after the compound is quantified in the acceptor well through analytical techniques, such as Liquid Chromatography–Mass Spectrometry (LC-MS) (Figure 1). This method allows characterizing the effective P*_e_* range of tested compounds [72].

He and colleagues (2020) developed a noteworthy real-time PAMPA assay by introducing a modification to the standard method. This approach enabled the detection of direct fluorescence using a fluorescent artificial receptor, comprising an encapsulated fluorescent dye and a macrocycle, which was incorporated into the acceptor chamber. This innovative method not only facilitates the quantification of permeability but also distinguishes between slow and fast diffusion across the membrane, making it a valuable tool for drug discovery, particularly for prodrugs susceptible to hydrolysis reactions [73].

Several examples using PAMPA assays have been reported for prodrugs, for example, the angiotensin II receptor antagonist candesartan [74] and oseltamivir [75,76].

### 2.4. Cell-Based Models

The cell-based permeability assessment model is widely utilized due to its ability to evaluate not only passive diffusion but also active transport, paracellular permeability, and efflux mechanisms. Among these models, the Caco-2 and Madin–Darby Canine Kidney (MDCK) cell lines are the most employed (Figure 1) [77]. Caco-2 cells exhibit the morphology of intestinal epithelium, while MDCK cells resemble distal tubule epithelium. Another significant difference lies in the types of transport systems. For Caco-2 cells, the most well-known systems include OATP1B1, OATP1B3, OATP2B1, PepT1, OCT1, OCT2, and OCT3. In contrast, for MDCK cells, the most recognized transporters are OCT2, peptides, and monocarboxylic acids. Efflux transporters can also vary, with Caco-2 cells predominantly expressing P-gp, MRP2, MRP4, and BCRP, while MDCK cells commonly express Mdr1, Mrp1, Mrp2, and Mrp5 [78].

The cell-based model experiment is relatively straightforward and involves plating the cells onto a porous support until they reach confluence, covering the surface of the device. For Caco-2 cells, confluence is achieved after 21 days, whereas for MDCK cells, it takes only 3–4 days. The tested compounds are added in a buffered saline solution to the apical surface of the cell layer, and after permeation, they are quantified in the basolateral collecting chamber using analytical methods [29,78,79,80,81].

Another possibility, by altering the order of addition into the basolateral layer, allows for the assessment of permeability through transporters (Basolateral to Apical Experiment: Ba-Ap). If the values obtained are the same, passive diffusion is the predominant process; however, if they differ, transport systems are likely involved in the permeability of the test compound [72]. Some limitations of the cell model are related to the low solubility of compounds and potential cytotoxicity, which may lead to inaccurate apparent permeability (P_app_) results.

**Figure 2:**
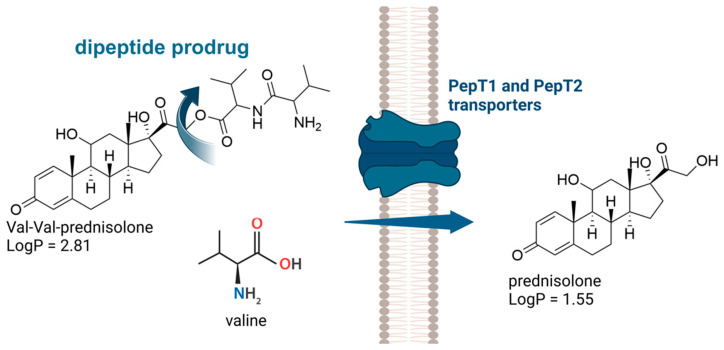
Prednisolone peptide prodrug. Adapted from [82] (Figure created in https://BioRender.com).

For example, Shen and colleagues synthesized dipeptide prodrugs of prednisone for ocular use, aiming to increase water solubility, enhance permeability, and reduce efflux by P-gp. The authors targeted the PepT1 and PepT2 transport systems, utilizing the valine–valine (Val-Val) sequence conjugated to prednisone and valine (Val) conjugated to prednisone to avoid P-gp-mediated efflux. It was demonstrated that the solubility of the Val–prednisone (4.24 mg/mL) and Val-Val–prednisone (3.11 mg/mL) prodrugs was significantly higher than that of the parental drug prednisone (0.31 mg/mL), while also exhibiting good chemical stability. The dipeptide prodrug (Val-Val–prednisone) (Figure 2) showed a half-life of 27 h at pH 7.4 and did not exhibit cytotoxic effects in MDCK cells at concentrations ranging from 5 to 250 μM. Unlike the parent corticosteroid, the Val-Val dipeptide prodrug was not a substrate for P-gp and was recognized by the peptide transport system in MDCK cells [82]. A similar strategy involving dipeptides to enhance drug permeability was described for several drugs, including acyclovir [83], lopinavir [84], and decitabine [85].

### 2.5. Other Methods to Determine the Permeability of Prodrugs

Several other methods have been used to evaluate the permeability of prodrugs, such as in situ perfusion, ex vivo gut sacs, and ex vivo diffusion chambers [86]. The in situ perfusion method allows the determination of prodrug permeability using the intact intestine, commonly through a single-pass approach, although recirculating methods have also been described [87,88,89,90]. In the single-pass method, a cannula is inserted into a section of the intestine of an anesthetized animal, and an aqueous solution containing the test compound is infused, allowing the determination of the effective permeability (P_eff_) calculated from the difference between the injected concentration and the collected concentration. Despite its use in animal models, the method has disadvantages, such as the need to standardize the flow rate, equilibrium time, intestinal region used, animal species, feeding status, solution osmolarity, and pH, among other factors (Figure 1) [88,89,90]. The in situ perfusion method can also be utilized to evaluate compound permeability across the BBB [91,92,93].

**Figure 3:**
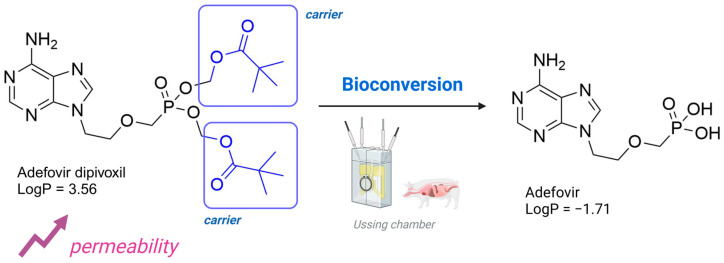
Adefovir prodrug. Adapted from [102] (Figure created in https://BioRender.com).

The everted gut sac model is another experimental approach for evaluating permeability, in which a section of the intestine, removed from an experimental animal, is everted over a tube. In this model, intestinal sacs of up to 4 cm are tied and immersed in a buffer solution containing the test substance. The concentration of the test compound is then measured using analytical methods [94,95,96,97]. The ex vivo diffusion chamber method involves extracting a segment of the intestine from an animal and placing it into a chamber containing donor and acceptor solutions. This method can also be used to evaluate the permeability of prodrugs [98]. Such a method was used to evaluate the permeability of phenytoin prodrugs by using excised porcine nasal mucosa assembled in an Ussing chamber [99]. Several experiments using ex vivo diffusion chamber methods have been reported elsewhere, highlighting their application [15,100,101]. Annaert and colleagues (2000) reported that the adefovir dipivoxil prodrug (Figure 3) exhibited an 100-fold increase in permeability by using in vitro models. This significant increase in permeability is the result of the functionalization of hydroxyls to form the methyl pivalate ester, which acts as a plasma carrier (Figure 3). However, the authors did not find the same correlation when using Ussing chambers, which highlights the importance of combining different permeability methods to characterize the effects of prodrugs [102].

## 3. Examples of Market Prodrugs Designed to Enhance Drug Permeability

To comprehend the impact of the prodrug approach to modify physical–chemical properties, all logP values reported in the figures within this subchapter were calculated using in silico methods with MarvinSketch 24.3.0, 2024, ChemAxon software (www.chemaxon.com).

In the context of passive transport, the diffusion of drugs through the membrane is facilitated by the absence of charges in the molecular structure. As a result, charged compounds typically exhibit lower permeability compared to neutral ones [103]. This challenge can be addressed by masking the molecular charge by carrier groups in prodrugs. Several prodrugs available on the market have been developed using this strategy [17].

Dimethyl fumarate (DMF) (1) (Table 2), for instance, is a hygroscopic powder with antifungal properties, which is often used as a dehumidifying agent to prevent contamination and deterioration. In 1959, Schweckendiek explored the use of DMF for psoriasis, drawing attention to the therapeutic potential of this simple molecule [104]. Today, DMF is approved not only for the treatment of psoriasis but also for multiple sclerosis. The drug exerts a pleiotropic effect on various targets, including erythroid-derived factor 2 (Nrf2), hydroxycarboxylic acid receptor 2 (HCAR2), and factor nuclear kappa B (NF-κB), among others [105]. Monomethyl fumarate (2) is the active form; however, the presence of the charged carboxylic acid at physiological pH limits its absorption through biological membranes via passive diffusion. To address this issue, the dimethyl derivative improves lipophilic parameters, thereby enhancing absorption [106]. After oral administration, DMF is rapidly bioconverted by esterases into monomethyl fumarate (2), the active metabolite, with a half-life of 36 h, while DMF itself has a half-life of 12 min [107].

The discovery of oseltamivir (3) (solubility: 82 mg/mL; logP: 1.2) serves as an excellent example of the successful application of the prodrug approach to enhance membrane permeability. In 2009, the H1N1 swine flu affected approximately 60 million individuals in the United States, resulting in an estimated 12,000 fatalities [108]. Oseltamivir (3) is an antiviral medication that functions by inhibiting the viral neuraminidase, an enzyme that cleaves sialic acid to prevent the release of viral particles. Due to the conserved nature of the neuraminidase active site, the drug demonstrates efficacy against various viral subtypes, with inhibitory concentration (IC_50_) values ranging from 0.01 to 69.2 nM [109]. Structure-based drug design focused on the neuraminidase active site was instrumental in the development of the compound GS 4071, which has an IC_50_ value of 2 nM against H1N1 and water solubility of 100 mg/mL. However, despite its effectiveness in inhibiting the viral enzyme, GS 4071 exhibited low bioavailability (~5%) in rats^43^, which prompted further molecular optimization to address the charged carboxylic acid that contributed to its low permeability. The ethyl ester prodrug successfully led to the development of oseltamivir (3), which demonstrated improved bioavailability in mice (30%), dogs (70%), and humans (80%) [109,110,111]. In vivo, human carboxylesterase 1 (CES1) hydrolyzes the ethyl ester, converting it into the active form [112,113].

Mofetil mycophenolate (4) (solubility: 43 mcg/mL; log P: 2.68) (Table 2) is an immunosuppressive prodrug used to prevent allograft rejection, which acts by inhibiting the enzyme inosine monophosphate dehydrogenase (IMPDH). It is about five times more potent to inhibit IMPDH type II isoforms expressed in lymphocytes than type I isoforms expressed in various cells, and thus interferes in the de novo synthesis of guanosine [114]. Although active, the inferior oral bioavailability of the parental mycophenolic acid (5) motivated the carboxylic acid masking to enhance membrane permeability. After oral administration of mofetil mycophenolate (4), the levels of mycophenolic acid (5) in plasma ranged from 80.7 to 94% [115,116]. Differently from the acid character of mycophenolic acid (5) that is ionized in plasma, mofetil mycophenolate (4) exhibits a slightly basic (pKa 5.7) and more neutral profile that favors its membrane diffusion by passive transport [117]. After oral administration of mofetil mycophenolate (4), it undergoes fast presystemic bioconversion through the CES-1 and CES-2 [118].

Dabigatran etexilate (6) (Table 2) serves as a notable example of a double-prodrug strategy [119]. This compound marks a significant advancement in the treatment of venous thromboembolism, achieving FDA approval more than 60 years after the introduction of traditional anticoagulants such as warfarin [120]. Classic anticoagulants, including warfarin and heparin, function as indirect thrombin inhibitors. Warfarin acts by inhibiting vitamin K epoxide reductase, while heparin exerts its effects by targeting specific clotting factors, both of which disrupt the blood coagulation cascade [120]. In contrast, dabigatran etexilate (6) demonstrated superior thromboprophylaxis and safety in randomized clinical trials, offering improved outcomes over conventional treatments for vascular events [121,122].

The active compound, dabigatran (7) (Table 2), is a reversible competitive inhibitor of thrombin, which was developed through studies on the crystal structure of the bovine thrombin–benzamidine complex [123]. However, dabigatran’s two ionizable groups result in poor cellular permeability. By incorporating hydrophobic ethyl ester and hexyloxycarbonyl carbamide chains, dabigatran etexilate (6), a double-prodrug, was created to enable oral absorption. Upon first-pass metabolism via hepatic and pancreatic carboxylesterases, the prodrug is converted into the active form, dabigatran (7) (Table 2), which exhibits a Ki of 4.5 nM. This active compound forms a stable complex with thrombin, effectively inhibiting its role in the coagulation cascade [120,124].

Capecitabine (8) (logP 1.43) (Table 2) is a carbamate derivative of the metabolically active 5-fluorouracil (9), representing another excellent example of prodrug development aimed at enhancing bioavailability. The oral formulation of capecitabine has rendered the intravenous administration of 5-fluorouracil largely obsolete [125]. Capecitabine is a antimetabolite agent indicated for various malignancies, including early-stage triple-negative breast cancer [126], pancreatic cancer [127], colorectal cancer [128], and biliary tract cancer [129]. Capecitabine (8) undergoes biotransformation in the liver, where carboxylesterases convert it to 5′-deoxy-5-fluorouridine (5′-DFUR). This intermediate is subsequently metabolized to 5-fluorouracil (5-FU) (9) (Table 2) by cytidine deaminase and thymidine phosphorylase (dThdPase), enzymes that are overexpressed in many tumor types. Due to this series of three metabolic steps, capecitabine is classified as a pre-prodrug of 5-FU [130].

While β-lactam antibiotics have profoundly transformed human history since the discovery of penicillin (10), the growing emergence of resistance poses significant global concerns [131,132,133]. Among the various mechanisms of resistance, the expression of the enzyme β-lactamase is the most prevalent and significant determinant of bacterial resistance to β-lactams, particularly in Gram-negative bacteria [134]. This is especially concerning as the rapid spread of resistance mechanisms threatens to render β-lactams, currently the most widely used drugs for treating bacterial infections, completely obsolete [135,136,137,138,139]. The enzyme β-lactamase catalyzes the hydrolytic cleavage of the β-lactam ring, thereby neutralizing the antibacterial activity of several drug classes, including penicillins, cephalosporins, and carbapenems [140]. Serine β-lactamases, a common subclass of β-lactamase enzymes, can be effectively inhibited by compounds such as clavulanic acid, tazobactam, and sulbactam. However, metallo-β-lactamases (MBLs), which utilize zinc ions in their active site for catalysis, are not susceptible to inhibition by these drugs. This resistance to conventional inhibitors makes MBL-producing bacteria a particular concern in clinical settings [141].

The prodrug strategy in β-lactams has been used in many ways to improve PK/PD properties and resist the action of β-lactamases. The problem of instability at acidic pH inherent to the penicillin penem core was the first challenge faced. The high susceptibility to hydrolysis of the β-lactam ring limits its oral absorption, which motivated the development of aminopenicillins, such as ampicillin (11) and amoxicillin (12) (Table 2). The presence of the primary amine group at the β-carbon of the carbonyl, in addition to increasing the stability of β-lactams at stomach pH, generates an analogous structure of tripeptides that increases absorption mediated by peptide transporters present in the enterocyte membrane [142]. However, the presence of an acid carboxyl group present in different β-lactam cores also penalizes the oral bioavailability of aminopenicillins. With this information in mind, the classical application of ester prodrugs was used to develop drugs with better permeability, such as pivampicillin (13), talampicillin (14), bacampicillin (15), hetacillin (16), and lenampicillin (17) (Table 2). All these prodrugs are extensively cleaved by bacterial esterases and release the active metabolite [143].

Sofosbuvir (SOF) (18) (Table 2) is a low-molecular-weight prodrug used in the treatment of chronic hepatitis C virus (HCV) infections. After oral administration, SOF (18) is rapidly metabolized into its active form, uridine triphosphate (GS-461203), through a series of enzymatic reactions, with phosphorylation being the key process for inhibiting viral replication (Table 2). The conversion of SOF (18) into its active metabolite (26) is essential for its efficacy, as the triphosphate directly inhibits HCV RNA polymerase. The permeability of SOF (25) across biological membranes is influenced by its solubility, which is pH-dependent and can affect its bioavailability. In vitro studies have shown that co-administration with ledipasvir (LDV), an NS5A protein inhibitor of HCV, significantly increases SOF (25) permeability, resulting in a 2.3-fold increase in plasma exposure. This interaction helps optimize therapeutic efficacy, as increased exposure may improve clinical outcomes. Additionally, the half-life of SOF (25), approximately 12 h, allows for convenient dosing and facilitates treatment adherence, particularly in prolonged regimens, as it reduces the need for frequent administration. The pharmacokinetic profile of SOF (25), with its efficient conversion to the active triphosphate, is a well-established option for the treatment of HCV [144].

Tenofovir (TFV) (19) (Table 2) is a nucleoside analogue widely used in the treatment of HIV infection, primarily acting as a competitive inhibitor of reverse transcriptase, thereby preventing the reverse transcription of viral RNA into DNA and inhibiting HIV replication. As a prodrug, TFV (19) undergoes intracellular conversion to its active form, tenofovir diphosphate (TFV-DP) (20), through a phosphorylation process catalyzed by cellular kinases, predominantly in T lymphocytes and macrophages (Table 2). This conversion to TFV-DP (20) is critical for its antiviral activity, as TFV-DP (20) is incorporated into the growing viral DNA strand, resulting in chain termination and inhibition of viral replication. Tenofovir disoproxil fumarate (TDF) (21) and tenofovir alafenamide (TAF) (22) are prodrugs of TFV (19), differing in their chemical structures and the efficiency with which they are converted to TFV (19) in target cells (Table 2). TDF (21) is converted into TFV (19) through hydrolysis of the disoproxil ester group, followed by the formation of TFV-DP (20). In contrast, TAF (22) is more efficiently metabolized within cells, releasing TFV (19) directly into target cells, thus allowing for lower oral doses of equivalent TFV (19) to be administered. This results in reduced plasma concentrations of TFV (27), minimizing systemic exposure to off-target organs such as the kidneys and reducing the renal toxicity associated with TDF (20). Furthermore, TAF (22) does not rely on renal transporters for cellular uptake, which further enhances its safety profile, particularly regarding renal toxicity. In terms of in vitro activity, TFV (19) exhibits an effective concentration (EC_50_) against HIV-1 of approximately 5.0 µM. In comparison, TDF (29) and TAF (20) demonstrate significantly greater potency, with EC_50_ values of 0.05 µM and 0.005 µM, respectively [145].

Adefovir dipivoxil (23) (Table 2) is a prodrug used in the treatment of viral infections, particularly hepatitis B and HIV (Table 2). Upon administration, the prodrug undergoes hydrolysis of the pivaloyloxymethyl (POM) ester group, converting it into adefovir, which is subsequently phosphorylated by cellular kinases to form adefovir diphosphate (PMEA-DP) (24) (Table 2). This active metabolite (24) is then incorporated into the growing viral DNA strand during replication, leading to chain termination and inhibition of viral replication. This phosphorylation process, critical for the drug’s antiviral activity, predominantly occurs in lymphocytes and hepatocytes, which are the primary target cells for the virus. The presence of the POM moiety in adefovir dipivoxil (23) plays a crucial role in enhancing the drug’s lipid solubility, which in turn improves its permeability across biological membranes. This structural modification allows the prodrug (23) to efficiently enter target cells, ensuring higher intracellular concentrations of the active form, adefovir diphosphate (24). Additionally, the co-crystallization of adefovir dipivoxil with carboxylic acids has been shown to improve its stability, which is beneficial in preventing degradation under challenging environmental conditions and enhancing overall bioavailability. Although adefovir dipivoxil (31) demonstrates improved permeability and stability compared to its active form, adefovir diphosphate (32), its renal excretion remains a concern. The drug and its metabolites are primarily eliminated through the kidneys, which may contribute to renal toxicity, especially with prolonged use. Therefore, careful monitoring of renal function is essential to optimize therapeutic outcomes while minimizing adverse effects. The reduced plasma concentrations of adefovir, achieved by using the prodrug, help mitigate systemic exposure to off-target organs, reducing the potential for toxicity [146,147].

Fosphenytoin (25) (Table 2) is a prodrug of phenytoin (26), developed to address the poor solubility and erratic absorption of phenytoin when administered through conventional routes. Its phosphate ester formulation enhances water solubility, enabling safer and more efficient intravenous and intranasal administration, particularly in emergency situations such as the management of epileptic seizures and status epilepticus. The conversion of fosphenytoin (25) to phenytoin (26) (Table 2) occurs rapidly in a concentration-dependent manner, facilitated by the activity of alkaline phosphatases present in the nasal mucosa and other tissues. This biotransformation process ensures the efficient release of the active drug, bypassing first-pass metabolism and thereby enhancing therapeutic efficacy [99].

### 3.1. Prodrugs to Enhance Ocular, Skin, and CNS Permeability

Tafluprost (27) (Table 3) is a fluorinated ester analogue of prostaglandin F_2α_ (28) used to treat ocular hypertension and open-angle glaucoma. Due to its lipophilicity as an ester, tafluprost (27) quickly penetrates the cornea, followed by hydrolysis to its active form, tafluprost acid (Table 3), by carboxyl esterase. The peak of plasmatic concentration peaks is achieved after 10 min of administration and falls below 10 pg/mL after 30 min. It may be used in monotherapy but achieves greater intraocular pressure reduction in conjunction with timolol [148,149]. It binds almost exclusively to the FP receptor, activating uveoscleral outflow and reducing intraocular pressure. In comparison to other prostaglandin analogs, latanoprost and travoprost, the binding affinity of tafluprost (27) towards FP is 12- and 2.8-fold higher, respectively [150].

Ophthalmic epinephrine (29) (Table 3) is used to reduce intraocular pressure in the treatment of open angle glaucoma [151]. Uveoscleral outflow is activated once epinephrine binds to α2 adrenergic receptors [152]. However, epinephrine’s (29) reduced lipophilicity makes its corneal penetration difficult, resulting in undesirable side effects like irritation, eyebrow pain and adrenochrome deposits in the cornea and conjunctiva [153]. Esterification of its phenolic hydroxyl group makes it 100 to 600 times more lipophilic than its parent drug, increasing corneal permeability and nearly eliminating the side effects. After penetration, dipivefrin (30) is hydrolyzed by esterase enzymes, releasing epinephrine (29) [151]. Both epinephrine (29) and dipivefrin (30) are absorbed locally, followed by systemic absorption [154]. Between 55% and 65% of the initially administered ophthalmic doses of these drugs are absorbed into systemic circulation (Table 2) [154].

While generally safe, cataract surgery can induce inflammation and adverse effects, such as elevated intraocular pressure and cystoid macular edema (CME), potentially reducing long-term visual acuity [155]. Consequently, topical NSAIDs are used in post-operative care, reducing ocular pain, inflammation, and CME. In comparison to NSAIDs like ketorolac, bromfenac, and flurbiprofen, nepafenac (31) is most likely to improve post-operative care [156]. Nepafenac (31) is a prodrug derived from amfenac (32), where the polar charge-bearing carboxyl group is converted to an amide, increasing its lipophilicity and corneal penetration [157]. Once nepafenac is bioactivated by hydrolases, the resulting amfenac inhibits Cyclooxygenase-1 (COX-1) and Cyclooxygenase-2 (COX-2) enzymes, halting arachidonic acid cascade and its inflammatory effects (Table 2) [158].

Tazarotene (42) is a synthetic retinoid approved to treat acne vulgaris, plaque psoriasis, and photoaging (Table 2) [159]. Additionally, it has been displayed to be efficient in the treatment of basal cell carcinoma [160], in situ squamous cell carcinoma [161], and ichthyosis [162], among others. Tazarotene (33) is administered topically, its lipophilic, uncharged nature makes its absorption quick in the lipid rich stratum corneum. Once it reaches the keratinocytes in the epidermis, it is quickly metabolized by esterases into its active form, tazarotenic acid (34), which selectively binds to the retinoic acid receptors (RARs) RAR-β and RAR-γ. Binding results in modulation of three pathogenic factors, reducing inflammation, normalizing keratinocyte differentiation, and reducing skin cell hyperproliferation (Table 2) [163]. Less than 6% of tazarotenic acid (34) is absorbed into the bloodstream, leading to minimal plasma concentrations and reduced systemic absorption [164].

Betamethasone dipropionate (35), a glucocorticoid prodrug, is administered topically by itself or in conjunction with calcipotriol in the treatment of psoriasis (Table 3). The parental drug, betamethasone (36) (Table 3), binds on the glucocorticoid receptors, then the ligand–receptor pair acts as a transcription factor or enhances other transcription factors, modulating gene expression (Table 2). Anti-inflammatory genes, such as DUSP1, GILZ, and ANXA1, are transactivated, while transrepressing pro-inflammatory transcription factors, such as NF-κB and AP-1. Consequently, inflammation and immune response due to inflammation, the causative factors of psoriasis, are reduced [165]. The prodrug (35) is a doubly esterified form of the parent drug (36); its increased lipophilicity facilitates stratum corneum penetration and once it reaches the dermis it is hydrolyzed, releasing the parent drug [166]. In a phase II clinical trial of a betamethasone dipropionate (35) containing cream with a concentration of 0.644 mg/g, about 27 subjects received a weekly average dose of 79 g of the cream. After four weeks, 24 subjects had undetectable betamethasone dipropionate plasma levels and three of them had levels ranging from 13 to 31 pg/mL, indicating low systemic absorption [167,168].

**Figure 4:**
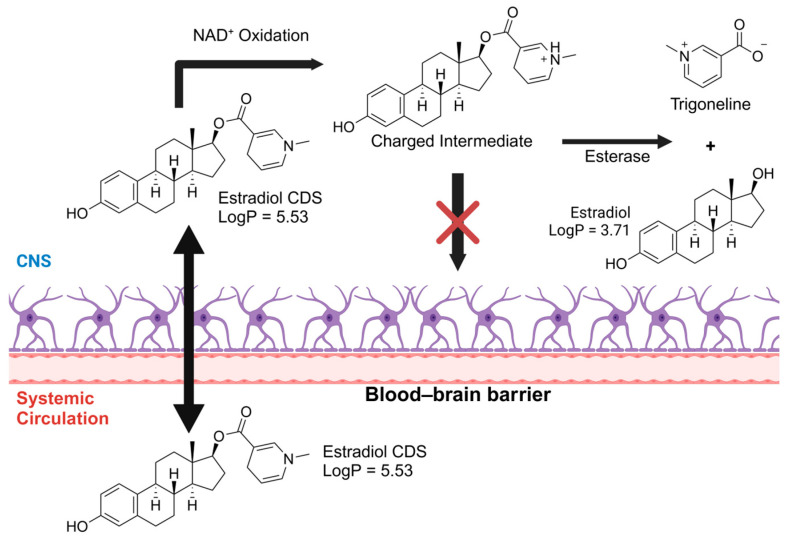
Estradiol prodrugs exploring a CDS to enhance BBB permeability. Adapted from [169] (Figure created in https://BioRender.com).

To improve BBB permeability, a more elaborate prodrug approach is needed, as is the case of Chemical Delivery Systems (CDSs), a prodrug approach with multiple bioactivating steps. Such an approach was employed for an estradiol CDS prodrug consisting of a dihydrotrigonelline moiety esterified to an estradiol molecule (Figure 4) [169]. The CDS would benefit from increased lipophilicity (logP 4.5) from the esterification and cross the BBB, followed by oxidation of the dihydrotrigonelline moiety to trigonelline, which is positively charged. Once the CDS acquires a charge, its lipophilicity dramatically decreases (logP −0.17), making it unable to cross the BBB again and getting trapped inside the CNS, where it lingers long enough to be slowly hydrolyzed, releasing estradiol locally [169,170]. Unfortunately, this CDS, known as Estredox or IDR-90104, had its phase II clinical trials discontinued [171].

Levodopa (37) (Table 3) is one of the few approved CNS targeting prodrugs designed to increase BBB permeability to treat Parkinson’s disease (Table 2). It is taken orally and absorbed in the intestine; however, enzyme *L*-amino acid decarboxylase (AADC) quickly metabolizes it and only 30% of the initial dose reaches systemic circulation. To avoid presystemic metabolism, levodopa is taken in combination with an AADC inhibitor, which is either benserazide or carbidopa; in this manner, oral bioavailability is nearly tripled [172]. Fasting absorption is rapid, reaching peak plasma concentrations after 15 to 60 min [173]. Once it reaches the BBB, levodopa is taken in through LAT1 [174] and once it reaches the CNS, it is bioactivated by AADC, releasing dopamine (38) [175]. The synthesized dopamine aims to replenish the deficit caused by substantia nigra degeneration [176].

The prodrug strategy applied to codeine (39) (Table 3), like most approved prodrugs targeting the CNS, does not improve its BBB permeability but rather its permeability in the gastrointestinal tract, due to increased lipophilicity resulting from hydroxyl masking [177]. After oral administration, codeine (39) is quickly absorbed, followed by peak plasma concentrations after 1 h, with a half-life of 3 to 3.5 h. It may also be administered intramuscularly, resulting in a faster plasma peak after 0.5 h [178]. Codeine (39) is metabolized into its parent drug, morphine (40), by cytochrome p450 enzyme, CYP2D6 [178]. In turn, morphine (40) crosses the BBB and binds as an agonist to the μ-opioid receptor, providing an analgesic effect (Table 2) [179].

### 3.2. The Use of Drug Transporters in the Pharmaceutical Market

The transport of pharmaceuticals and endogenous molecules across cellular membranes is mediated by drug transporters, which are integral membrane proteins crucial for the pharmacokinetic processes of drugs. These transporters facilitate the movement of drugs and other substances via active transport, utilizing the energy derived from ATP hydrolysis or through ion or concentration gradients [180]. Drug transporters are typically classified into three major superfamilies: ATP-Binding Cassette (ABC), Solute-Linked Carrier (SLC), and Solute Carrier Organic Anion (SLCO). The role of these transporters in facilitating the movement of molecules is particularly significant in drug development, where their interaction with therapeutic agents can have profound implications for drug efficacy and safety [22,181]. In prodrug design, structural scaffolds can be strategically tailored to target transporters expressed in specific tissues, enhancing pharmacokinetic properties and minimizing off-target effects. After crossing the membrane, the prodrug undergoes cleavage within the cytoplasm, releasing the active drug. Several transporters have been explored to improve drug permeability including: transporters of amino acids, oligopeptides, carbohydrates, bile acid, vitamins, and carnitine [182].

The ABC superfamily predominantly consists of primary active uniporters, which employ ATP hydrolysis to translocate molecules unidirectionally across cellular membranes [183]. The SLC superfamily comprises secondary active transporters that utilize electrochemical or concentration gradients to mediate the transport of drugs and ions and may also harness the energy derived from ion gradients generated by ATP-dependent pumps [184]. The SLCO superfamily, previously categorized under the SLC21 subfamily and now recognized as Organic Anion Transporting Polypeptide (OATP), is responsible for the transport of larger molecules, such as xenobiotics and bile acids, and plays a critical role in the absorption and elimination of both endogenous and exogenous compounds. These superfamilies exemplify the diversity of transport mechanisms, each with distinct roles in optimizing the absorption and disposition of drugs, which is crucial for the development of effective drug delivery systems [22]. This transporter-mediated transport is essential for the ADME of drugs, significantly influencing their therapeutic efficacy and safety profiles within the body [180,181,182,183,184,185,186,187].

Among the widely used transport systems, the amino acid transporter LAT1/SLC7A5 stands out. LAT1 is a non-glycosylated protein that forms a complex with the membrane protein 4F2hc/SLC3A2, and is expressed in several cells of the central nervous system and also in brain tumors such as gliomas [188]. Several drugs are known to use this transport system to cross the BBB, including gabapentin, levodopa, baclofen, methyldopa, pregabalin, and melphalan. The design of prodrugs exploiting this transport system usually involves conjugation of the drug with the amino acids phenylalanine or tyrosine [184].

Similarly, valacyclovir (42) (Table 4), an ester prodrug of acyclovir (41), utilizes transporter systems such as human peptide transporter-1 (hPEPT-1) to enhance the bioavailability of acyclovir, which is limited by its low intestinal absorption (Table 3). Upon oral administration, valacyclovir (42) is absorbed in the gastrointestinal tract via specific peptide transporter proteins, including hPEPT-1, which is responsible for the active transport of dipeptides and structurally similar compounds, such as valacyclovir (42), which is esterified with the amino acid *L*-valine. This interaction with transporters underscores the central role of transporter-mediated absorption in overcoming bioavailability limitations of certain drugs [189].

The esterification of acyclovir (41) (Table 4) with *L*-valine increases valacyclovir’s (23) affinity for hPEPT-1 and potentially other peptide transporters, such as human intestinal peptide transporter-1 (HPT-1). This interaction with high-capacity transporters facilitates its translocation across the intestinal epithelium, resulting in significantly higher bioavailability compared to acyclovir administered orally. After absorption, valacyclovir (42) undergoes hydrolytic cleavage by esterase enzymes in intestinal and hepatic tissues, rapidly releasing acyclovir (41), the pharmacologically active molecule. The improved bioavailability of valacyclovir (42) mirrors the benefits observed with XP13512, as both prodrugs enhance the systemic availability of their active components through transporter-mediated mechanisms [189].

The improved bioavailability of valacyclovir (42) enables the dosing frequency and dosage to be reduced compared to acyclovir (41), which optimizes patient compliance. Furthermore, the efficient conversion and enhanced transport of valacyclovir (42) contribute to elevated plasma concentrations of acyclovir (52), thereby improving its therapeutic efficacy in the treatment of herpes zoster, genital herpes, and as a prophylactic treatment for cytomegalovirus in immunocompromised patients [190].

In the context of neurological treatments, the role of drug transporters is also evident in the action of valbenazine (54) (Table 4), which is a selective inhibitor of the vesicular monoamine transporter 2 (VMAT2). Valbenazine (54) is a valine ester of the active metabolite [+]-α-dihydrotetrabenazine ([+]-α-HTBZ) (55), which, after metabolic conversion, exerts most of the pharmacological activity of the compound (Table 3) [191]. This metabolite has high affinity for VMAT2, with significant selectivity for VMAT2 over off-target receptors, minimizing the risk of unwanted interactions. Pharmacokinetically, valbenazine (54) has a half-life of 15 to 20 h and is administered once daily, with stable pharmacokinetics unaffected by food intake [191,192,193].

Several studies in the literature highlight the design of prodrugs exploring conjugation with transporter substrates to increase drug permeability. This strategy is a powerful tool to explore in drug design aiming to enhance permeability across the membranes [19,85,191,192,193,194,195,196].

## 4. New Technologies in Prodrugs to Enrich Permeability Properties

### 4.1. Prodrugs of PROteolysis TArgeting Chimeras (PROTACs)

With the advances in understanding the human genome, it has become possible to estimate that protein-coding genes may give rise to more than 20,000 proteins that could serve as potential therapeutic targets [197]. However, despite this knowledge, many of these potential targets remain challenging to address, such as transcription factors. In this regard, new intervention strategies must be developed, such as PROTACs. The term PROTAC was first introduced over 20 years ago by Crews and colleagues. This innovative approach leverages heterobifunctional molecules designed to degrade specific target proteins. These molecules bind to a protein of interest (POI), facilitating its ubiquitination and subsequent degradation via the proteasome [198].

Since the first PROTAC was reported by the Crews group, it has been noted that, despite its effectiveness in protein degradation and low cytotoxicity, challenges such as low chemical stability, solubility, permeability, and failure to comply with Lipinski’s rule of five (Ro5) may impact its oral bioavailability [199,200].

Typically, strategies for optimizing the properties of PROTACs to improve permeability and solubility involve modifications in the linker chains. Small linkers containing polar and/or ionizable groups can help achieve an appropriate balance of physicochemical properties, thereby enhancing these physicochemical properties [201]. For solubility, for example, it has been proposed that the use of values superior to 60 μg/mL seems to be reasonable as a starting point [202]. The prediction of solubility for PROTACs is a difficult task due to molecular conformation and chameleonicity issues related to the PROTACs, but some authors have proposed that BRlogD and TPSA values with threshold values of 2.58 and 289 Å^2^ could be desirable for soluble PROTACs [201]. Nevertheless, due to the requirement for recognition by the POI, it is also necessary to establish conformational restrictions to ensure the correct geometry of the ternary complex. This can, however, lead to an increase in the lipophilicity of PROTACs, making it more challenging to maintain a proper balance of physicochemical properties [200]. In such situations, incorporating rigid linkers with cationic subunits can help improve permeability. Moreover, exploring strategies to enhance intracellular uptake, such as the use of carriers like cell-penetrating peptides that are recognized by membrane receptors, holds significant promise for addressing the challenges of low permeability. Additionally, the prodrug approach presents an interesting and effective strategy to overcome the limitations of PROTACs, particularly with respect to solubility and permeability.

**Figure 5:**
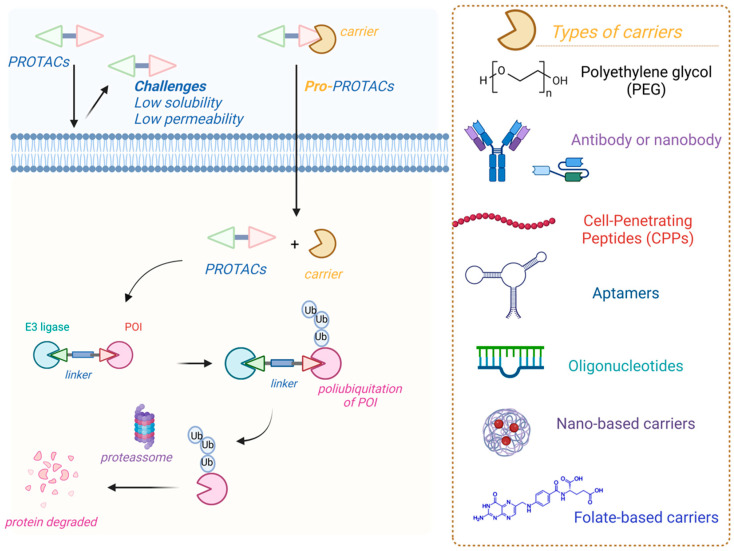
Strategies exploring prodrug approaches to improve PROTAC permeability. Adapted from [205] (Figure created in https://BioRender.com).

Recently, the concept of prodrug PROTACs (pro-PROTACs) has emerged as an alternative that may optimize these compounds, enabling selective delivery to target tissues while minimizing off-target effects [203]. Controllable PROTACs have been thoughtfully designed through various innovative approaches, such as light activation, photocaging, and photoswitchable systems, as well as conjugation with peptides, aptamers, and folate (Figure 5). Moreover, strategies like hypoxia activation, radiotherapy-triggering, and reactive oxygen species-responsiveness have also been explored and described in the literature [204].

**Figure 6:**
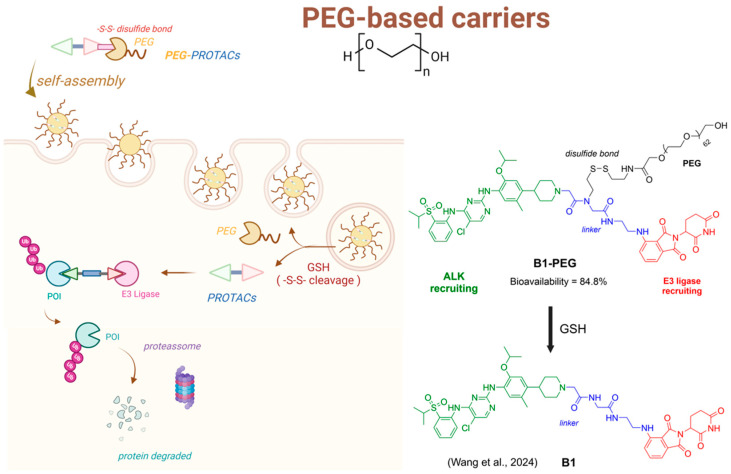
PEG-based prodrugs to enhance PROTAC permeability [206]. (Figure created in https://BioRender.com).

Wang and colleagues (2024) explored a novel strategy to enhance the permeability of PROTACs by designing an amphiphilic PROTAC incorporating a long-chain polyethylene glycol (PEG) linked to a disulfide bond, which acts as a glutathione-responsive subunit, allowing for selective cleavage inside tumor cells. The authors focused on degrading the echinoderm microtubule-associated protein-like 4-anaplastic lymphoma kinase (EML4-ALK), a protein implicated in several cancers, including non-small cell lung cancer (NSCLC). By using this protein as the target, they employed ceritinib as the ligand and pomalidomide for cereblon (CRBN) recognition by the E3 ligase. PEG was chosen as the linker due to its hydrophilic properties, and the amphiphilic nature of the PROTAC led the authors to hypothesize that the pro-PROTAC could self-assemble into micelles in aqueous environments, significantly improving its solubility. Given the high glutathione levels in tumor cells, the disulfide bond would be reduced and cleaved, facilitating PROTAC release within tumor cells. The authors demonstrated the antiproliferative effects of the PROTAC through ALK degradation. In vivo, the pro-PROTAC B1-PEG exhibited an impressive absolute bioavailability of 84.8%, compared to 63.5% for the conventional PROTAC. Moreover, studies using the H3122 xenograft mouse model demonstrated the superior efficacy of the pro-PROTAC over both the conventional PROTAC and ceritinib, highlighting the enhanced physicochemical properties and cell permeability of the pro-PROTAC (Figure 6) [206].

**Figure 7:**
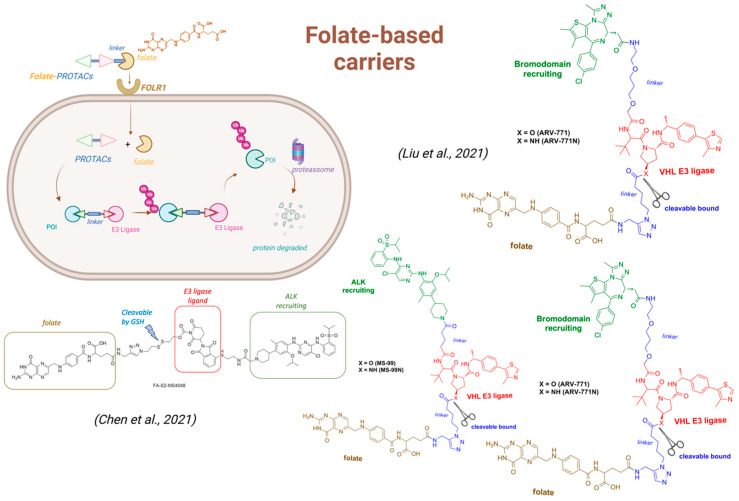
Folate-based carriers are designed as pro-PROTAC to enhance permeability [205,208]. (Figure created in https://BioRender.com).

The incorporation of folate as a carrier can improve drug uptake, thereby enhancing the permeability of anticancer agents. The use of folate as a carrier group to improve permeability and facilitate uptake by tumor cells has been extensively applied in the design of novel antitumor agents. In tumors, three folate uptake systems have been identified: folate receptors (FRs), the reduced folate carrier (RFC), and the proton-coupled folate transporter (PCFT) [207]. The incorporation of folate as a carrier can improve drug uptake, thereby enhancing the permeability of anticancer agents. In a compelling study conducted by Liu and colleagues, the use of folate as a carrier for PROTACs was thoughtfully investigated. The authors conjugated the von Hippel–Lindau (VHL) E3 ubiquitin ligase to four distinct PROTACs, aiming to degrade BRDs (compound ARV771-N), MEKs (MS-432N), and ALK (compound MS99N) (Figure 7). Given the high expression of folate receptors in various cancers (such as breast, lung, and ovarian cancers), utilizing folate as a carrier significantly enhances uptake by tumor cells, facilitating the degradation of these overexpressed proteins. The study elegantly demonstrated that targeting folate receptor-1 (FOLR1) can greatly improve the permeability of PROTACs in tumor cells, while also reducing their potential toxicity [208].

In another study led by Chen and colleagues (2021), the use of folate was investigated to enhance the permeability and uptake of pro-PROTACs (Figure 7). The authors employed pomalidomide as a ligand for the CRBN E3 ligase and utilized a reduction-cleavable disulfide linker, which could be cleaved by glutathione inside the cell, enabling the release of the PROTAC named FA-S2-MS4048. Since GSH levels are relatively low in the bloodstream but significantly higher in the cytoplasm, this mechanism may offer a more selective release, potentially reducing toxicity to non-tumor cells. In this study, anaplastic lymphoma kinase (ALK) was chosen as the POI for the PROTAC, and MS4048 was employed as the ligand for ALK. The folate–disulfide linker–glutarimide conjugate was designed to minimize interaction with CRBN in other cells, enabling selective activation following disulfide reduction to release the PROTAC. Furthermore, through FOLR1 knockdown experiments using shRNA, the authors demonstrated that the degradation of the NPM-ALK fusion by FA-S2-MS4048 was absent, reinforcing that the pro-PROTAC targets FOLR1-positive cancer cells, emphasizing the critical role of folate as a carrier for the pro-PROTAC FA-S2-MS4048 [205].

The modulation of physicochemical properties in PROTACs presents several challenges, which have driven the exploration of innovative conjugation methodologies to enhance both permeability and selectivity of action. Notably, strategies such as utilizing aptamers, antibodies, peptides, or nano-based carriers have been highlighted in the literature as promising solutions for addressing issues related to permeability [209].

The conjugation of PROTACs with aptamers offers an innovative strategy to enhance tumor permeability while reducing off-target effects. Aptamers are single-stranded oligonucleotide sequences that can selectively bind to specific targets or proteins [209]. Since the FDA’s approval of pegaptanib (Macugen^®^) for macular degeneration in 2004, numerous clinical trials have investigated the therapeutic potential of these "chemical antibodies". Aptamers present several advantages over conventional antibodies, including lower immunogenicity and higher permeability through tissues [210]. Their precise target recognition, particularly in tumor cells, makes aptamers an intriguing carrier group to explore in the development of pro-PROTACs.

**Figure 8:**
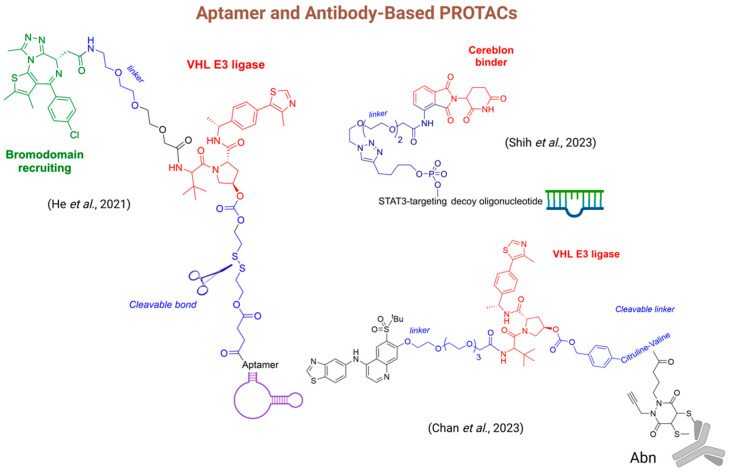
Aptamer and antibody-based PROTACs. [212,213,214] (Figure created in https://BioRender.com).

He and his colleagues (2020) have described the conjugation of the aptamer AS1411, which selectively binds to nucleolin, a protein highly expressed on tumor cell membranes (Figure 8) [211]. In this approach, the PROTAC utilized JQ-1 as the ligand for bromodomain (BRD4) and VHL as the E3 ligase ligand, connected by an ethoxylated spacer. A disulfide spacer was incorporated as a cleavage bond into the VHL ligand, and it was designed to be cleaved by endogenous glutathione. This linker was also coupled with the aptamers to enhance the uptake and permeability of the pro-PROTAC. Studies conducted in fetal bovine serum demonstrated high chemical stability, alongside significant cellular uptake, highlighting the improved permeability of the conjugate in comparison to conventional PROTACs. The antitumor effect in vivo was evaluated using an MCF-7 xenograft mouse model with intravenous administration at a 10 μM concentration. The conjugate showed superior in vivo antitumoral effects compared to both the PROTAC and aptamers alone [212].

With the aim of degrading nucleolin, Zhang and co-workers (2022) developed a combination of the PROTAC ZL226 with the aptamer AS1411. This combination enhances the permeability of cancer cells, improving selectivity towards these cells. The compound demonstrated its chemical stability, with a half-life of approximately 70.5 h [215]. The relatively small size of aptamers (30 kDa for a 100 bp ssDNA) compared to antibodies has been recognized as a significant advantage, allowing for more efficient penetration into cells and tissues, including the central nervous system [216]. In an insightful study by Shi and colleagues (2019), the permeability of two aptamers, GMT8 and Gint4.T, which target U87MG cells and PDGFRβ for glioblastoma treatment, was carefully examined. The researchers developed an in vitro model using U87MG and bEnd.3 cells seeded in transwell inserts. Their findings demonstrated the aptamers’ capacity to permeate through blood–brain barrier mimetic assays. Furthermore, they explored the complexation of the aptamers with paclitaxel, an anticancer drug known for promoting microtubule stabilization, which does not naturally cross the blood–brain barrier in an intact form. The aptamer–paclitaxel complex successfully facilitated paclitaxel’s cellular entry, suggesting that this aptamer transport system may offer valuable potential in enhancing drug permeability within the central nervous system [217].

Shih and colleagues (2023) investigated the conjugation of PROTACs with aptamers to target the degradation of the signal transducer and activator of transcription 3 (STAT3) (Figure 8). STAT3, a transcription factor identified in the 1990s, plays a crucial role in cancer progression but presents a significant challenge for small molecule interventions, as is often the case with transcription factors. The pro-PROTAC was thoughtfully designed by incorporating an oligonucleotide that binds to STAT3 (the POI) and pomalidomide as the cereblon ligand, linked by a spacer. The rationale behind this design is that cereblon, acting as an E3 ubiquitin ligase, would facilitate the ubiquitination and degradation of STAT3. The prodrug exhibited enhanced efficacy compared to pomalidomide or the STAT3-targeting decoy oligonucleotide alone, with a Degradation Concentration 50% (DC_50_) of 261 nM and a Growth Inhibition 50% (GI_50_) of 792 nM [213].

The use of antibody–drug conjugates to enhance the selectivity of cytotoxic agents has become a well-established approach in cancer chemotherapy. This strategy involves the binding of a monoclonal antibody to specific antigens on the surface of tumor cells, facilitating their internalization and the subsequent intracellular release of the cytotoxic agent. The choice of a spacer agent plays a crucial role in modulating the physicochemical and pharmacokinetic properties, thereby improving the conjugate’s half-life and permeability [218].

While conjugates provide notable benefits in selectively delivering cytotoxic agents, certain challenges remain in their design. These challenges, including limited penetration into the microenvironment of specific solid tumors, suboptimal payload potency, potential immunogenicity, emergence of resistance, and reduced chemical stability of the conjugate, highlight the need for exploring novel approaches [219]. Antibody–PROTAC conjugation presents a promising alternative for enhancing permeability while minimizing the use of agents that are highly cytotoxic to healthy cells.

While PROTACs represent a promising strategy for targeting the degradation of specific proteins, they often face challenges such as limited selectivity, variable permeability, and suboptimal pharmacokinetic properties for oral administration. These limitations can be addressed and improved through antibody conjugation [200]. Several studies have highlighted the promising potential of antibody–PROTAC conjugates, showcasing their ability to reduce toxicity, enhance selectivity and potency, and improve permeability [220,221,222]. This review does not aim to cover all examples of antibody–PROTAC conjugates used to enhance selectivity and permeability, as these can be explored in other articles [222,223,224,225]. Instead, we will provide an example of the application of this strategy developed by Chan and colleagues (2023). These authors developed novel PROTACs targeting the degradation of serine- and threonine-protein kinase 2 (RIPK2) in HER2+ cell lines. RIPK2 is a kinase implicated in various diseases, including cancer, inflammation, and multiple sclerosis. To achieve selectivity, the authors employed trastuzumab (Abn) as a monoclonal antibody, conjugating it to the PROTAC via a linker containing dibromopyridazinedione for the formation of a disulfide bridge with the antibody, and a cleavable valine–citrulline–para-aminobenzyl alcohol moiety (Figure 8). The conjugation yielded a drug-to-antibody ratio (DAR) of 4.0 for trastuzumab. A control conjugate anti-IL-4 with DAR 3.7 was also prepared to assess selectivity. In studies using the SKOV3 HER2+ ovarian cancer cell line, both the PROTAC and antibody–PROTAC demonstrated similar activity at 10 nM concentrations. However, the antibody–PROTAC offered the added advantage of acting specifically on HER2+ cells. Beyond selectivity, the antibody–PROTAC exhibited improved permeability due to selective internalization via endocytosis [214].

**Figure 9:**
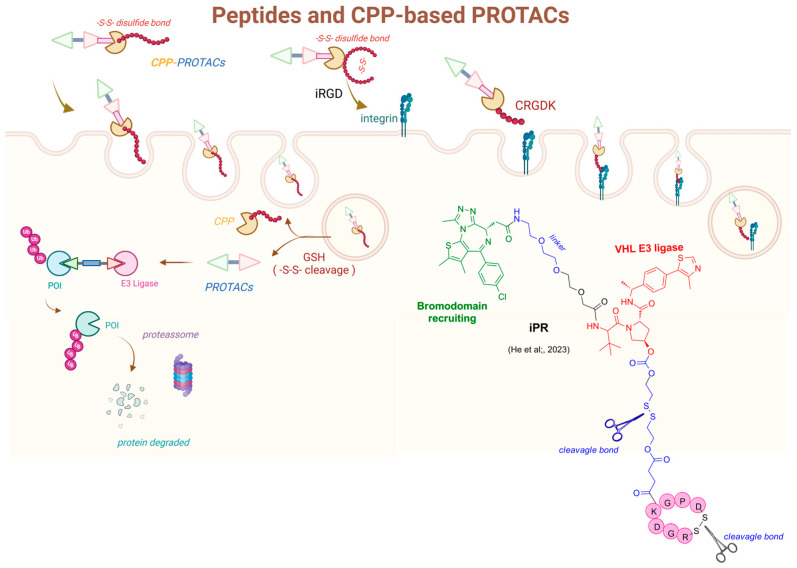
Peptide and CPP-based PROTACs [231] (Figure created in https://BioRender.com).

The use of peptides to enhance permeability and facilitate membrane crossing is a significant strategy in medicinal chemistry. The discovery that certain peptides, such as the HIV transactivator (TAT) factor and specific oligoarginines, possess the ability to traverse cell membranes and enter cells led to the development of the concept of CPPs (Figure 9) [226]. Typically, CPPs are small peptides composed of fewer than 40 amino acids that promote cellular entry through various mechanisms, including direct penetration through membranes and endocytosis. These peptides can be utilized as transport systems to improve compound permeability when conjugated as prodrugs, and commonly CPPs exhibit positive charge in physiological pHs due to the presence of lysine and arginine residues [227,228,229]. Predicting the cell-penetrating ability of peptides is a valuable tool in designing CPP sequences. Artificial intelligence models, such as artificial neural networks (ANNs), have been shown to offer high predictive accuracy (80–100%), making them a powerful resource in this planning process [230].

The use of peptides to enhance cell permeability holds significant potential for the development of new conjugates with PROTACs. Given the challenges associated with the low solubility, permeability, and bioavailability of PROTACs, peptide conjugation emerges as a promising strategy for improving their physicochemical properties and biological activity. He and colleagues initially employed a conventional approach utilizing VHL-based PROTACs to target BRD4 degradation. However, they encountered challenges with inadequate tumor penetration, which threatened to compromise the efficacy of the PROTACs in vivo. To overcome this obstacle, they innovated pro-PROTAC by integrating peptides containing the RGD integrin recognition motif (Figure 9). This design specifically targets αvβ3 integrin, a protein abundantly expressed in tumor cells. Upon administration, the RGD peptide (iRGD, CRGDKGPDC) undergoes cleavage, resulting in the formation of NRP1 recognition peptides (CRGDK). NRP1, a transmembrane protein present on the tumor surface, facilitates the entry of PROTACs into tumor cells, thereby enhancing permeability. This peptide conjugation notably improved the water solubility of the PROTACs, achieving values exceeding 10 mM, in contrast to the conventional PROTACs, which exhibited a solubility of 29.5 μM. Furthermore, the pro-PROTACs demonstrated commendable chemical stability in PBS containing 5% serum. In vivo experiments using a mouse model with MDA-MB-231 xenografts revealed that this peptide conjugation significantly enhanced antitumor effects without any adverse effects during treatment. Specifically, daily administration of 5 μM of conventional PROTACs for 21 days suppressed tumor growth by up to 36%, whereas the pro-PROTACs achieved a reduction of up to 62.3% at the same concentration. These findings underscore the potential of the prodrug approach in improving PROTAC permeability and efficacy [231].

Recently, Zhang and collaborators (2024) synthesized 26 novel peptides to improve permeability, utilizing lymphoma and leukemia cell models. Among these peptides, two—C9C-f(3Bta) and Cyclo-C9C–R—exhibited enhanced permeability and stability within the cellular environment. The researchers subsequently conjugated one of these peptides with a PROTAC designed to degrade the Bcr-Abl protein in K562 cells. Their findings revealed that peptide conjugation resulted in a substantial increase in the PROTAC’s permeability across the cell membrane, demonstrating the effectiveness of this approach for improving the cellular uptake of PROTACs [232].

Dai and collaborators (2022) employed a strategy of conjugating CPPs with PROTACs to enhance cell permeability and promote the degradation of programmed cell death protein 1 (PD-1). PD-1 plays a critical role in suppressing T-cell-mediated immune responses by binding to its primary ligand, PD-L1, which reduces immune activation. Elevated expression of PD-L1 has been linked to tumor cells evading immune responses, and its inhibition is considered a promising approach to improving cancer immunotherapy. To address this, the authors designed PROTACs targeting the degradation of PD-1 or PD-L1, conjugating them with peptides to enhance permeability in cervical cancer cell lines (C33A and HeLa). In their design, VHL was utilized as the E3-ligase ligand, and the peptide sequence “RRRRRRRR” was identified as particularly effective in improving PROTAC’s permeability [233]. Yokoo and colleagues (2021) conducted an intriguing study in which they developed a chimeric helix-stabilized peptide (stPERML) incorporating the CPP hepta-arginine to enhance the permeability of a PROTAC targeting estrogen receptor α, demonstrating the effectiveness of this approach in addressing the permeability challenges commonly associated with PROTACs [234].

### 4.2. PROTACs and Polymers

**Figure 10:**
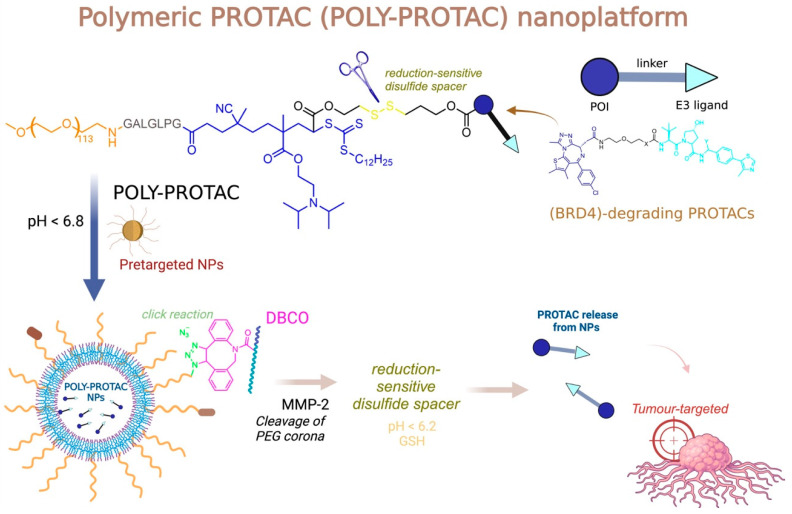
Polymeric PROTAC nanoplatform. Adapted from [237] (Figure created in https://BioRender.com).

An alternative and promising strategy to address the various limitations of PROTACs, such as off-target effects due to inadequate distribution in the body, low solubility, and limited permeability, involves the use of Nanosized Drug Delivery Systems (NDDSs) and polymer conjugation. In the first approach, encapsulating PROTACs within nanoparticulate systems enhances their stability and pharmacokinetic properties, including an extended half-life. Furthermore, NDDSs can facilitate tumor cell uptake by improving permeability through various mechanisms, such as receptor-mediated pathways and endocytosis. Nanoparticulate systems—including polymeric, lipid-based, metallic, dendrimeric, metal–organic frameworks (MOFs), and mesoporous silica—offer a valuable opportunity to advance the study of PROTACs [235]. Nanoparticulate systems often leverage molecular interactions between polymeric matrices and PROTACs during physical encapsulation. However, chemical conjugation with carriers and biomaterials presents a promising alternative, as it helps maintain the stability of the pro-PROTAC during transport while enabling selective activation in specific compartments. In polymer conjugation, the linkers employed can be triggered by external stimuli (e.g., magnetism, ultrasound, and radiation) or internal stimuli (e.g., hypoxia, acidic environment, and oxidative stress) [236]. A noteworthy example was presented by Gao and colleagues (2022), who designed a system in which bromodomain-containing protein 4 (BRD4)-degrading PROTACs were conjugated with an amphiphilic copolymer containing a disulfide bond. These polymer conjugates, referred to as POLY-PROTACs, demonstrated the ability to self-assemble into nanoparticulate micelles that respond to metalloproteinase-2 activity and the reductive tumor microenvironment, leading to the accumulation of POLY-PROTAC in tumor tissue (Figure 10). Once inside tumor cells, the disulfide bond is cleaved by glutathione (GSH), releasing the PROTAC to facilitate BRD4 degradation. Additionally, the authors further modified the nanoparticulate system to introduce an azide functional group, enabling click chemistry reactions with dibenzocyclooctyne (DBCO). This modified polymeric conjugate exhibited a 3.9-fold higher accumulation in tumor tissue compared to the original POLY-PROTAC, highlighting its potential for the selective delivery of pro-PROTACs to tumor sites [237].

**Figure 11:**
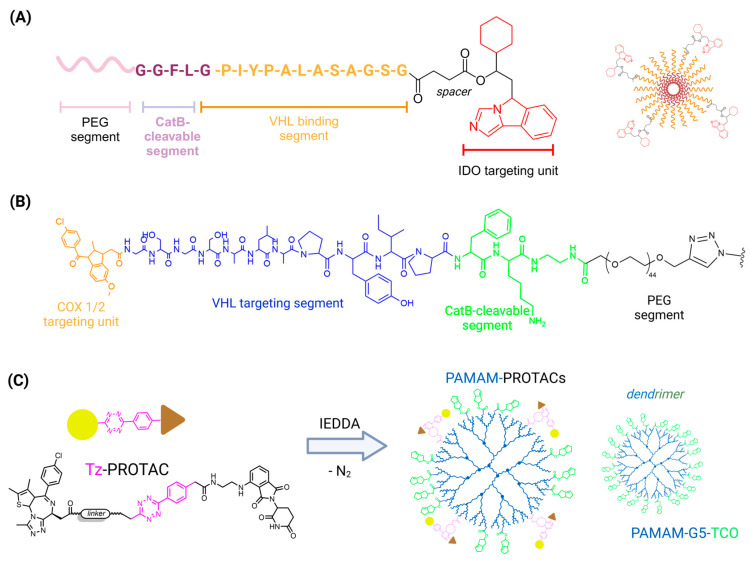
Polymeric PROTAC nanoplatform. (**A**) Semiconducting polymer nano-PROTAC [238]. (**B**) Smart nano-proteolysis targeting Chimeras [239]. (**C**) tetrazine-modified PROTACs and TCO-modified dendrimer [240]. (Figure created in https://BioRender.com).

Another example of PROTAC conjugation with polymers was reported by Zhang and colleagues (2021). In this study, the PROTAC was designed to include the von Hippel-Lindau protein (VHL) as the E3 ligase ligand and indoleamine 2,3-dioxygenase (IDO) as the protein of interest (POI), an enzyme involved in tryptophan catabolism that is overexpressed in tumor tissues and contributes to the suppression of dendritic cell activity. As the POI substrate, the previously described inhibitor NLG919 was employed. In this PROTAC, VHL and NLG919 was coupled through a succinic acid linker (Figure 11A). The VHL subunit was conjugated to a cathepsin B substrate, which is commonly overexpressed in solid tumors such as breast, colorectal, and prostate cancers. The cathepsin B substrate was further linked to an amphiphilic semiconducting polymer containing a PEG group. This system represents an intriguing approach that integrates the pro-PROTAC strategy with polymer conjugation, aiming to develop more efficient PROTAC delivery systems. In vivo experiments conducted on 4T1 tumor-bearing mice demonstrated that, 24 h after the administration of PROTAC-conjugated nanoparticles, a significant inhibition of tumor growth was observed [238].

In a subsequent study, the group synthesized and evaluated another smart nano-PROTAC, in which the indomethacin subunit was selected as a ligand for COX-1/2, the protein of interest for degradation. The rationale behind COX-1/2 degradation in tumor cells was to reduce prostaglandin E_2_ (PGE_2_) levels, as this molecule is known to promote immunosuppressive cells, potentially enhancing tumor immunogenicity. The PROTAC was designed with a COX-1/2 ligand (indomethacin) conjugated to a VHL-targeting segment (Figure 11B). Additionally, the authors incorporated a substrate for cathepsin B recognition and cleavage. As a carrier system, an amphiphilic self-assembling polymer—poly(cyclopentadithiophene-alt-benzothiadiazole) (PCB) conjugated to poly(ethylene glycol) (PEG, Mw = 2000)—was utilized to deliver the pro-PROTAC via click chemistry. The authors demonstrated COX-1/2 degradation through in vitro (90%) and in vivo (80%) experiments, resulting in up to 90% depletion of PGE2 [239]. This polymer conjugation strategy represents an innovative approach to reprogramming the immune response and reactivating it against tumors. Furthermore, it highlights how drug delivery conjugation strategies can help mitigate the challenges commonly associated with PROTACs.

This article does not intend to provide an exhaustive review of studies exploring the conjugation of PROTACs with polymers for nanosystem development; however, several noteworthy articles have already addressed this topic [239,241,242,243,244]. The examples presented in this work illustrate how the conjugation of PROTACs with excipients commonly used in formulation development can lead to pro-PROTACs with enhanced solubility and permeability. This serves as an important optimization tool, considering that, due to the intrinsic properties of PROTACs, they are often classified within the BCS system as Class II or IV, where permeability is generally moderate or low [245].

Another strategy to enhance permeability involves the use of dendrimers as carriers [246,247,248]. Dendrimers are highly structured polymeric macromolecules at the nanoscale, consisting of multiple branched monomers extending from a central core [248]. When used as carriers, drugs or bioactive compounds can be conjugated to dendrimers through various approaches, including interaction with a spacer, direct attachment to the dendrimer surface, or incorporation into the growing branches of the dendrimer [247].

The use of dendrimers can enhance the permeability of PROTACs while promoting event-driven degradation control. In an insightful study by Jin and colleagues (2023), a PAMAM dendrimer functionalized with trans-cyclooctene (TCO) was utilized to scavenge intracellular PROTACs containing a tetrazine-modified linker through a Diels–Alder reaction (Figure 11C). The authors referred to this strategy as “ligation to scavenging”, designed to disrupt event-driven degradation by using dendrimers [240].

### 4.3. Co-Crystal and Prodrugs

Co-crystals are defined as crystalline substances composed of two or more distinct molecular entities, typically active pharmaceutical ingredients (APIs) or APIs in combination with co-crystal formers. These entities are stabilized within the crystal lattice through various molecular non-covalent interactions, including Van der Waals forces, ionic interactions, hydrogen bonding, and hydrophobic interactions [249].

The formation of co-crystals is often explored to enhance solubility, while their use to improve permeability is less extensively described [250]. Despite that, both physic-chemical properties allow the combination of fixed-dose regimens to integrate components that act synergistically in several treatments. The development of co-crystals not only simplifies treatment by combining two active pharmaceutical ingredients (APIs) but also enhances patient adherence and therapeutic outcomes. Furthermore, the co-crystallization of two APIs can significantly improve bioavailability and stabilize certain drugs that may otherwise exhibit instability [251,252,253,254,255].

**Figure 12:**
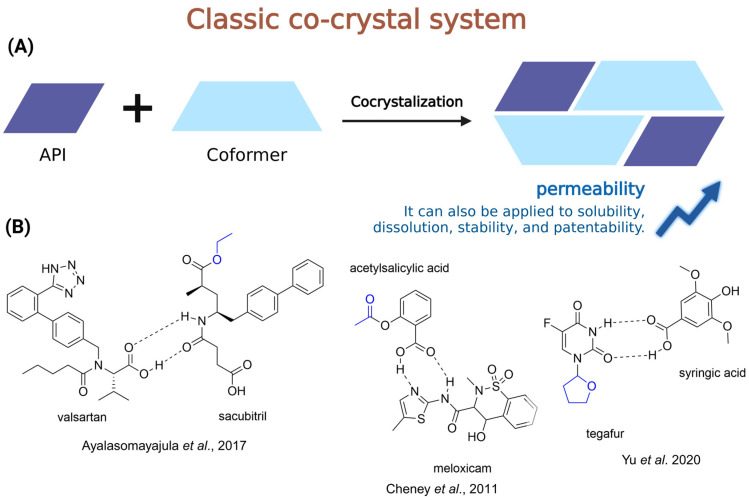
Co-crystal and prodrugs. (**A**) Classic co-crystal system, between active pharmaceutical ingredients (APIs) and conformer (**B**) Prodrug co-crystal examples [256,257,258] (Figure created in https://BioRender.com).

Entresto^®^, for example, is a medication developed by Novartis in 2015 for the treatment of heart failure (Figure 12). It is formulated as a co-crystal composed of valsartan and sacubitril in a 1:1 ratio. Sacubitril is a prodrug that functions as an inhibitor of neprilysin, an enzyme involved in the degradation of vasoactive substances such as brain natriuretic peptide (BNP) and other peptides. By inhibiting neprilysin, sacubitril contributes to lowering blood pressure, increasing natriuresis, and reducing afterload. Valsartan, in turn, acts as an angiotensin II type-1 (AT-1) receptor blocker, and its combination with sacubitril enhances antihypertensive effects [255]. Beyond their pharmacodynamic synergy, co-crystal formulation also offers advantages in improving the bioavailability of valsartan [256].

Another example of how co-crystal improved permeability was reported by Cheney and colleagues (2011), where meloxicam was co-crystallized with acetylsalicylic acid, leading to a fourfold increase in bioavailability and up to a 44-fold improvement in solubility (Figure 12) [257].

Yu et al. (2020) developed a co-crystal of the anticancer agent tegafur. This prodrug, which is converted in vivo into 5-fluorouracil, has low permeability and solubility, resulting in limited bioavailability and necessitating the administration of high doses. Syringic acid, a natural compound found in various plant species, was selected as the co-crystal former. The authors evaluated the intrinsic dissolution rate (IDR) of both tegafur and the co-crystal at pH levels 1.2, 4.0, and 6.8. At pH 6.8, for instance, the IDR values for tegafur and the co-crystal were 1.76 and 3.17 mg·min^−1^·cm^−2^, respectively. Furthermore, the co-crystal demonstrated a 4.14-fold increase in permeability compared to tegafur. Pharmacokinetic studies in rats indicate that the co-crystal exhibits higher bioavailability, and a longer half-life compared to the prodrug tegafur alone. The authors suggest that several factors may contribute to this enhancement, including improved solubility and dissolution rate, which increase the concentration gradient across the membrane and facilitate diffusion. Additionally, the logP of syringic acid is 1.04, whereas tegafur has a logP value of −0.27 (Figure 12). Consequently, the incorporation of these compounds into the co-crystal lattice enhances the hydrophobic character of the crystal, improving its membrane permeability [258]. Additional mechanisms have been suggested to explain the enhanced permeability induced by co-crystals, including their ability to reduce transepithelial electrical resistance (TEER) by influencing tight junctions between membranes, thereby facilitating drug diffusion [259].

Understanding how co-crystals, particularly those containing prodrugs, can enhance permeability and influence pharmacokinetic parameters presents a valuable opportunity for innovation.

## 5. Conclusions

This review article aims to summarize the seamless integration of computational and experimental methodologies in prodrug development. Methods to characterize drug permeability using advanced in silico tools, including ML-based predictive models and molecular dynamics simulations, are complemented by robust experimental platforms such as PAMPA assays, cellular models (e.g., Caco-2 and MDCK), and ex vivo techniques (e.g., everted gut sacs and diffusion chambers). These approaches enable comprehensive characterization of molecular properties, elucidating transport mechanisms and the role of specific carriers such as PepT1 and LAT1 in selective and efficient drug delivery.

Case studies of clinically significant prodrugs, including paclitaxel, oseltamivir, and dabigatran etexilate, underscore the transformative potential of rational molecular design. These examples demonstrate how structural modifications—such as masking carboxylic groups or incorporating hydrophilic moieties—enhance permeability, chemical stability, and bioavailability. The success of dual prodrug strategies, exemplified by dabigatran etexilate, further illustrates the capacity of strategic design to optimize pharmacokinetics, enabling precise activation of the parent drug at therapeutic targets.

The emergence of PROTACs represents a complementary advancement in targeted drug development. Unlike prodrugs, which rely on bioreversible chemical modifications to optimize pharmacokinetics and pharmacodynamics, PROTACs employ a bifunctional design to selectively degrade disease-related proteins via the ubiquitin–proteasome pathway. By addressing limitations in druggability and specificity, PROTACs expand the toolkit of medicinal chemistry, particularly for targets previously considered inaccessible. While distinct in mechanism, the integration of PROTACs into drug discovery paradigms aligns with the overarching goal of improving therapeutic efficacy and overcoming barriers related to solubility, permeability, and selective targeting.

The prodrug approach represents a pivotal strategy in modern medicinal chemistry, addressing critical limitations in biopharmaceutical and pharmacokinetic properties, such as permeability, solubility, and bioavailability. By leveraging chemically modified, bioreversible derivatives, this strategy provides a systematic solution to overcome barriers associated with drug absorption, distribution, and therapeutic efficacy, ultimately optimizing pharmacological profiles and safety margins.

In conclusion, the prodrug paradigm transcends conventional drug design by offering a robust, scientifically grounded framework for addressing pharmacokinetic and pharmacodynamic challenges. The integration of computational advancements, experimental rigor, and rational design principles provides an unparalleled pathway for innovation in drug development. As medicinal chemistry continues to evolve, the prodrug strategy will remain indispensable, particularly in overcoming complex challenges of bioavailability and therapeutic targeting, ensuring the development of safer, more effective treatments.

## Figures and Tables

**Table 1:** Biopharmaceutical Classification System (BCS).

Class	Solubility ^1^	Permeability ^2^	Examples of Drugs
I	High	High	Acyclovir, captopril, abacavir
II	Low	High	Atorvastatin, diclofenac, ciprofloxacin
III	High	Low	Cimetidine, atenolol, amoxicillin
IV	Low	Low	Furosemide, chlorthalidone, methotrexate

^1^ A compound is regarded as highly soluble when its therapeutic dose fully dissolves in 250 mL of an aqueous medium. ^2^ Likewise, a compound is classified as highly permeable if it demonstrates a bioavailability of ≥85%, indicating that at least 85% of the administered dose is recovered in the urine, considering phase 1/2 metabolites. The Biopharmaceutics Classification System (BCS) values are predetermined by the FDA. Adapted from [25,26].

**Table 2:** Marketed prodrugs.

Structure	Name (Code)	LogP ^1^	Current Development Stage
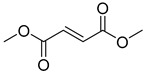	Dimethyl fumarate (1)	1.51	Market prodrug
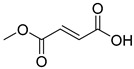	Monomethyl fumarate (2)	0.77	Drug
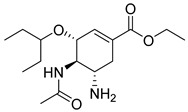	Oseltamivir (3)	1.23	Market prodrug
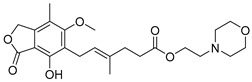	Mofetil mycophenolate (4)	2.68	Market prodrug
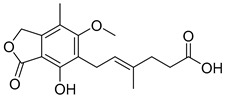	Mycophenolic acid (5)	3.01	Drug
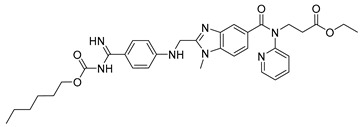	Dabigatran etexilate (6)	4.86	Market prodrug
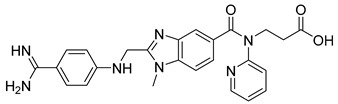	Dabigatran (7)	2.04	Drug
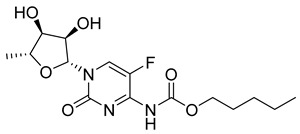	Capecitabine (8)	1.43	Market prodrug
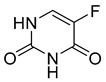	5-fluorouracil (9)	−0.08	Market prodrug
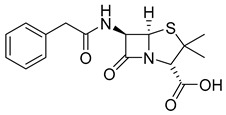	Penicillin (10)	0.93	Drug
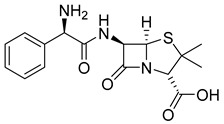	Ampicillin (11)	0.15	Drug
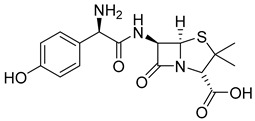	Amoxicillin (12)	−0.13	Drug
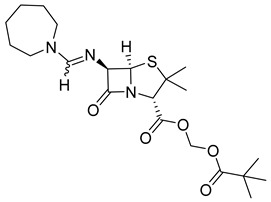	Pivmecillinam (13)	3.16	Market prodrug
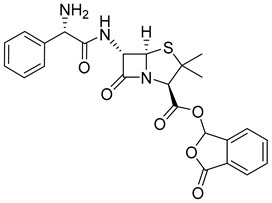	Talampicillin (14)	1.67	Market prodrug
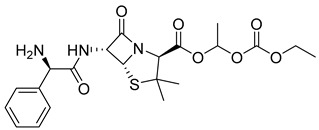	Bacampicillin (15)	1.22	Market prodrug
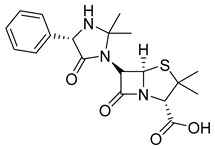	Hetacillin (16)	1.63	Market prodrug
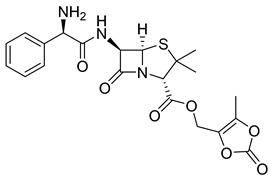	Lenampicillin (17)	0.97	Market prodrug
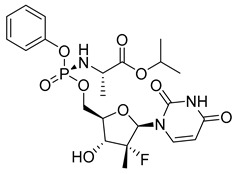	Sofosbuvir (18)	1.81	Market prodrug
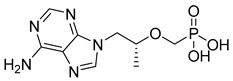	Tenofovir (19)	−1.30	Drug
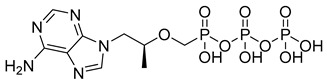	Tenofovir diphosphate (20)	−1.84	Market prodrug
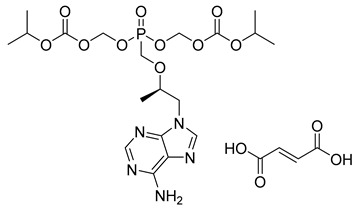	Tenofovir disoproxil fumarate (21)	3.02	Market prodrug
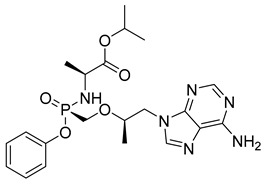	Tenofovir alafenamide (22)	2.05	Market prodrug
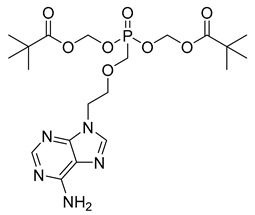	Adefovir dipivoxil (23)	3.56	Market prodrug
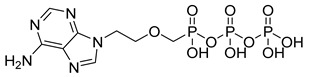	Adefovir diphosphate (24)	−2.28	Market prodrug
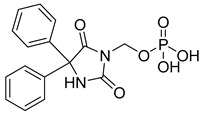	Fosphenytoin (25)	1.81	Market prodrug
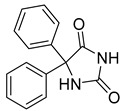	Phenytoin (26)	2.28	Drug

^1^ LogP values calculated by ChemAxon MarvinSketch.

**Table 3:** Marketed prodrugs to enhance ocular, skin, and CNS permeability.

Structure	Name (Code)	LogP ^1^	Current Development Stage
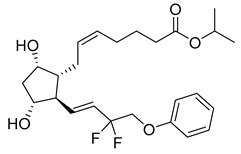	Tafluprost (27)	4.11	Market prodrug
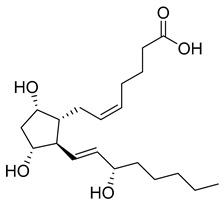	Prostaglandin F2a (28)	2.20	Drug
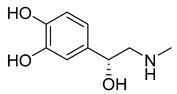	Epinephrine (29)	0.33	Drug
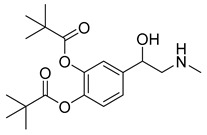	Dipivefrin (30)	3.61	Market prodrug
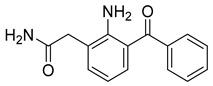	Nepafenac (31)	1.90	Market prodrug
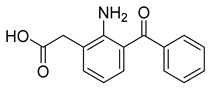	Amfenac (32)	2.77	Drug
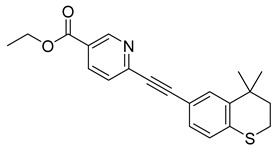	Tazarotene (33)	5.30	Market prodrug
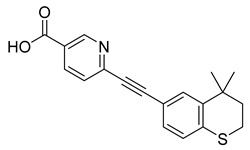	Tazarotenic acid (34)	4.33	Drug
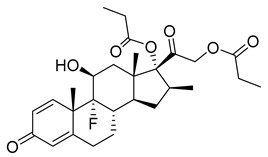	Betamethasone dipropionate (35)	3.86	Market prodrug
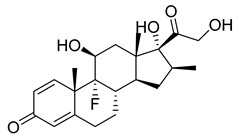	Betamethasone (36)	1.75	Drug
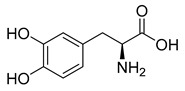	Levodopa (37)	0.58	Drug
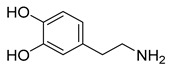	Dopamine (38)	0.85	Active metabolite
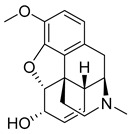	Codeine (39)	1.27	Market prodrug
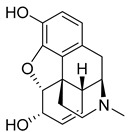	Morphine (40)	1.23	Drug

^1^ LogP values calculated by ChemAxon MarvinSketch.

**Table 4:** Marketed prodrugs that leverage drug transporter systems.

Structure	Name (Code)	LogP ^1^	Current Development Stage
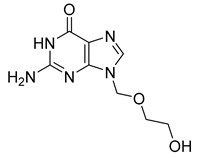	Acyclovir (41)	−1.85	Drug
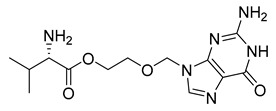	Valacyclovir (42)	−0.92	Market prodrug
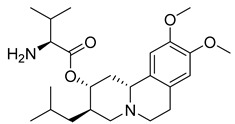	Valbenazine (43)	3.29	Market prodrug

^1^ LogP values calculated by ChemAxon MarvinSketch.

## Data Availability

Data sharing is not applicable.

## Abbreviations

5′-DFUR	5′-deoxy-5-fluorouridine
5-FU	5-fluorouracil
AADC	L-amino acid decarboxylase
AAMD	All-atom MD
ABC	ATP-Binding Cassette
ACh	Acetylcholine
AChE	Acetylcholinesterase
ACV	Acyclovir
ADEPT	Antibody-directed enzyme prodrug therapy
ADME	Absorption, distribution, metabolism, and excretion
ALK	Anaplastic lymphoma kinase
ANNs	Artificial neural networks
API	Adenosine triphosphate
AT1	Active pharmaceutical ingredients
ATP	Angiotensin II type-1
AUC	Area under the curve
BBB	Blood–brain barrier
BCS	Biopharmaceutical Classification System
BNP	Brain natriuretic peptide
BRD4	Bromodomain-containing protein 4
CDSs	Chemical delivery systems
CES1	Carboxylesterase 1
CGMD	Coarse-grained MD
CME	Cystoid macular edema
CNS	Central nervous system
COX-1	Cyclooxygenase-1
COX-2	Cyclooxygenase-2
CPPs	Cell-penetrating peptides
CRBN	Cereblon
DAR	Drug-to-antibody ratio
DBCO	Dibenzocyclooctyne
DC_50_	Degradation Concentration 50%
deuHTBZ	Deuterated dihydrotetrabenazine isomers
DL	Deep learning
DMF	Dimethyl fumarate
dThdPase	Cytidine deaminase and thymidine phosphorylase
*E. coli*	*Escherichia coli*
EC_50_	Effective concentration
EML4-ALK	Echinoderm microtubule-associated protein-like 4-anaplastic lymphoma kinase
ESBLs	Extended-spectrum β-lactamases
FDA	Food and Drug Administration
FOLR1	Folate receptor-1
FRs	Folate receptors
GI_50_	Growth Inhibition 50%
Gly	Glycine
GSH	Glutathione
HCAR2	Hydroxycarboxylic acid receptor 2
HCV	Chronic hepatitis C virus
His	Histidine
HIV	Human immunodeficiency virus
hPEPT-1	Human peptide transporter 1
IC_50_	Inhibitory concentration
IMPDH	Inosine monophosphate dehydrogenase
LASSO	Least Absolute Shrinkage and Selection Operator
LAT1	l-type amino acid transporter
LC-MS	Liquid Chromatography–Mass Spectrometry
LDV	Ledipasvir
Leu	Leucine
LogD	Logarithm of the distribution coefficient
LogP	Logarithm of the octanol/water partition coefficient
MBLs	Metallo-β-lactamases
MD	Molecular dynamics
MDCK	Madin–Darby Canine Kidney
ML	Machine learning
MOFs	Metal–organic frameworks
NDDSs	Nanosized Drug Delivery Systems
NF-κB	Factor nuclear kappa B
Nrf2	Erythroid-derived factor 2
NSAID	Non-steroidal anti-inflammatory drugs
NSCLC	Non-small cell lung cancer
OATP	Organic Anion Transporting Polypeptide
PAMPA	Parallel Artificial Membrane Permeability Assay
Papp	Apparent permeability coefficient
PBPs	Penicillin-binding proteins
PCFT	Proton-coupled folate transporter
PD-1	Cell death protein 1
Pe	Permeability coefficient
P_eff_	Effective permeation
PEG	Polyethylene glycol
PGE_2_	Prostaglandin E_2_
POI	Protein of interest
POM	Pivaloyloxymethyl
PROTACs	PROteolysis TArgeting Chimeras
PS-1	Presenilin-1
RAFT	Reversible Addition Fragmentation chain Transfer
RFC	Reduced folate carrier
RIPK2	Serine- and threonine-protein kinase 2
Ro5	Rule of five
SLC	Solute-Linked Carrier
SLCO	Solute Carrier Organic Anion
SOF	Sofosbuvir
STAT3	Signal transducer and activator of transcription 3
TAF	Tenofovir alafenamide
TEER	Transepithelial electrical resistance
TFV	Tenofovir
TFV-DP	Tenofovir diphosphate
Tyr	Tyrosine
Val	Valine
VHL	von Hippel–Lindau protein
VMAT2	Vesicular monoamine transporter 2
WHO	World Health Organization
